# Systematic identification of Oct4 transcriptional targets in embryonic stem cells using the auxin-inducible degron system and nascent RNA sequencing

**DOI:** 10.1186/s13619-025-00269-3

**Published:** 2025-12-03

**Authors:** Yuting Yuan, Dongmei He, Mingqiang Deng, Ying Ye, Peixin Chen, Hao Wu, Jie Zhang, Xichen Bao, Xiwei Wang, Wensheng Zhang

**Affiliations:** 1 Department of Biochemistry and Molecular Biology, Binzhou Medical University, Yantai, 264003 China; 2 Peninsula Cancer Research Center, Binzhou Medical University, Yantai, 264003 China; 3 Center for Cell Lineage Atlas, China-New Zealand Joint Laboratory On Biomedicine and Health, Guangdong Provincial Key Laboratory of Stem Cell and Regenerative Medicine, Guangdong-Hong Kong Joint Laboratory for Stem Cell and Regenerative Medicine, GIBH-CUHK Joint Research Laboratory On Stem Cell and Regenerative Medicine, State Key Laboratory of Respiratory Disease, Guangzhou Institutes of Biomedicine and Health, Chinese Academy of Sciences, Guangzhou, 510530 China; 4 University of Chinese Academy of Sciences, Beijing, 100049 China; 5 Department of Basic Research, Guangzhou National Laboratory, Guangzhou, 510005 China; 6 Department of Clinical Pathobiology and Immunological Testing, School of Medical Laboratory, Qilu Medical University, Zibo, 255300 China; 7 Cam-Su Genomic Resource Center, Medical College of Soochow University, Suzhou, 215123 China; 8 Key Laboratory of Biological Targeting Diagnosis, Therapy and Rehabilitation of Guangdong Higher Education Institutes, The Fifth Affiliated Hospital, Guangzhou Medical University, Guangzhou, 510700 China; 9 School of Life Sciences and Medicine, Shandong University of Technology, Zibo, 255049 China

**Keywords:** Mouse embryonic stem cells (mESCs), Oct4, Auxin-inducible degron (AID) system, Nascent RNA sequencing, Transcription targets

## Abstract

Oct4 is a key transcription factor essential for maintaining pluripotency and self-renewal in embryonic stem cells (ESCs), where it activates pluripotency-related genes and represses differentiation-associated genes. While previous studies have identified OCT4 target genes using methods such as chromatin immunoprecipitation sequencing (ChIP-seq) and RNA interference (RNAi), these approaches may not fully capture direct transcriptional regulation. The auxin-inducible degron (AID) system, which enables rapid and reversible protein degradation, combined with nascent RNA sequencing, provides a refined method for identifying direct transcriptional targets by detecting immediate transcriptional changes in both protein-coding genes and non-coding RNAs. In this study, we utilized mouse Oct4-mAID ESCs and nascent RNA sequencing with 5-ethynyl uridine (5-EU) labeling to systematically identify direct OCT4 targets. Our results uncovered novel potential OCT4 targets, providing a dataset for further research into the functions and regulatory networks of Oct4 and related transcription factors.

**Supplementary Information:**

The online version contains supplementary material available at 10.1186/s13619-025-00269-3.

## Background

Embryonic stem cells (ESCs) possess the unique abilities of unlimited self-renewal and pluripotency, allowing them to differentiate into all somatic cell lineages, thereby serving as a powerful model for studying early development and regenerative medicine applications (Elkenani and Mohamed 2022; Young 2011). The core transcription factors Oct4, Nanog and Sox2 form a central regulatory network that is essential for maintaining the pluripotent state of ESCs and supporting their self-renewal (Boyer et al. 2005; Chambers and Tomlinson 2009; Loh et al. 2006). Among these, Oct4 is pivotal, functioning as a gatekeeper of pluripotency by activating key genes associated with the pluripotent state, such as *Fgf4*, *Utf1*, *Rex*1 and *Opn*, while repressing genes linked to lineage commitment, including *Cdx2* and *Hand1* (Christophersen and Helin 2010; Niwa 2001). Recent evidence also indicates that phase separation of OCT4 contributes to the reorganization of topological-associated domains (TADs), thereby facilitating cell fate transitions (Wang et al. 2021b). Disruption of Oct4 expression results in ESC differentiation, underscoring its role in preserving an undifferentiated state, which is critically dependent on maintaining Oct4 levels within a narrow optimal range (Niwa et al. 2002; Niwa et al. 2000; Zafarana et al. 2009).

Efforts to define the direct targets and regulatory pathways of Oct4 have historically relied on techniques such as chromatin immunoprecipitation sequencing (ChIP-seq) (Boyer et al. 2005; Kim et al. 2008; Liu et al. 2008), RNA interference (RNAi) (Chen et al. 2008; Loh et al. 2006) or knockout (KO) (Shin et al. 2022). Although ChIP-seq identified thousands of Oct4-binding sites, evolutionary conservation analysis revealed fewer than 100 conserved targets, with enrichment in pathways involved in cell adhesion and motility (Livigni et al. 2013). These findings highlight the need for more refined approaches to precisely map the direct transcriptional targets of Oct4.

The auxin-inducible degron (AID) system provides an innovative solution for studying protein function through rapid and reversible protein degradation (Natsume et al. 2016; Nishimura et al. 2009). This approach involves tagging the target protein with an AID motif and co-expressing the auxin receptor OsTIR1 from Oryza sativa. The addition of auxin promotes the interaction between the AID-tagged protein and the SCF ubiquitin ligase complex, resulting in ubiquitination and proteasomal degradation of the target protein (Nishimura et al. 2009; Prozzillo et al. 2020; Tan et al. 2007). Compared to traditional gene knockout techniques, the AID system enables temporal control of protein depletion, minimizing irreversible genetic changes and reducing off-target effects associated with incomplete silencing (Jackson et al. 2003; Kaelin 2012). As such, it has become a powerful tool for dissecting the functions of key pluripotency factors in ESCs (Atkins et al. 2020; Ng et al. 2019; Nora et al. 2017).

Conventional RNA sequencing (RNA-seq), which typically focuses on mature polyadenylated RNA, can miss rapid transcriptional changes and unstable transcripts that are crucial for understanding immediate regulatory responses (Gallego Romero et al. 2014; Xiong et al. 2019; Xu and Asakawa 2019). In contrast, nascent RNA sequencing captures newly synthesized RNA, providing a direct measure of transcriptional activity (Wissink et al. 2019). This approach is particularly advantageous for detecting subtle, transient changes in transcription, including the expression of unstable non-coding RNAs such as enhancer RNAs, thereby offering a deeper insight into complex regulatory processes related to cellular growth, development, disease, and metabolic signaling (Muhar et al. 2018). By combining the AID system with nascent RNA sequencing, it is possible to identify direct transcriptional targets of Oct4 with higher temporal resolution.

In this study, we employed an Oct4-mAID mouse ESC line (Li et al. 2022) and nascent RNA sequencing via 5-ethynyl uridine (5-EU) labeling (Palozola et al. 2021) to systematically capture transcriptional changes during the rapid degradation of the OCT4 protein. This integrative approach enabled the identification of novel direct OCT4 targets, expanding the current understanding of its regulatory network and providing a valuable dataset for future research into the functions of Oct4 and associated transcriptional mechanisms.

## Results

### Functional verification of Oct4-mAID ESCs

**Fig. 1:**
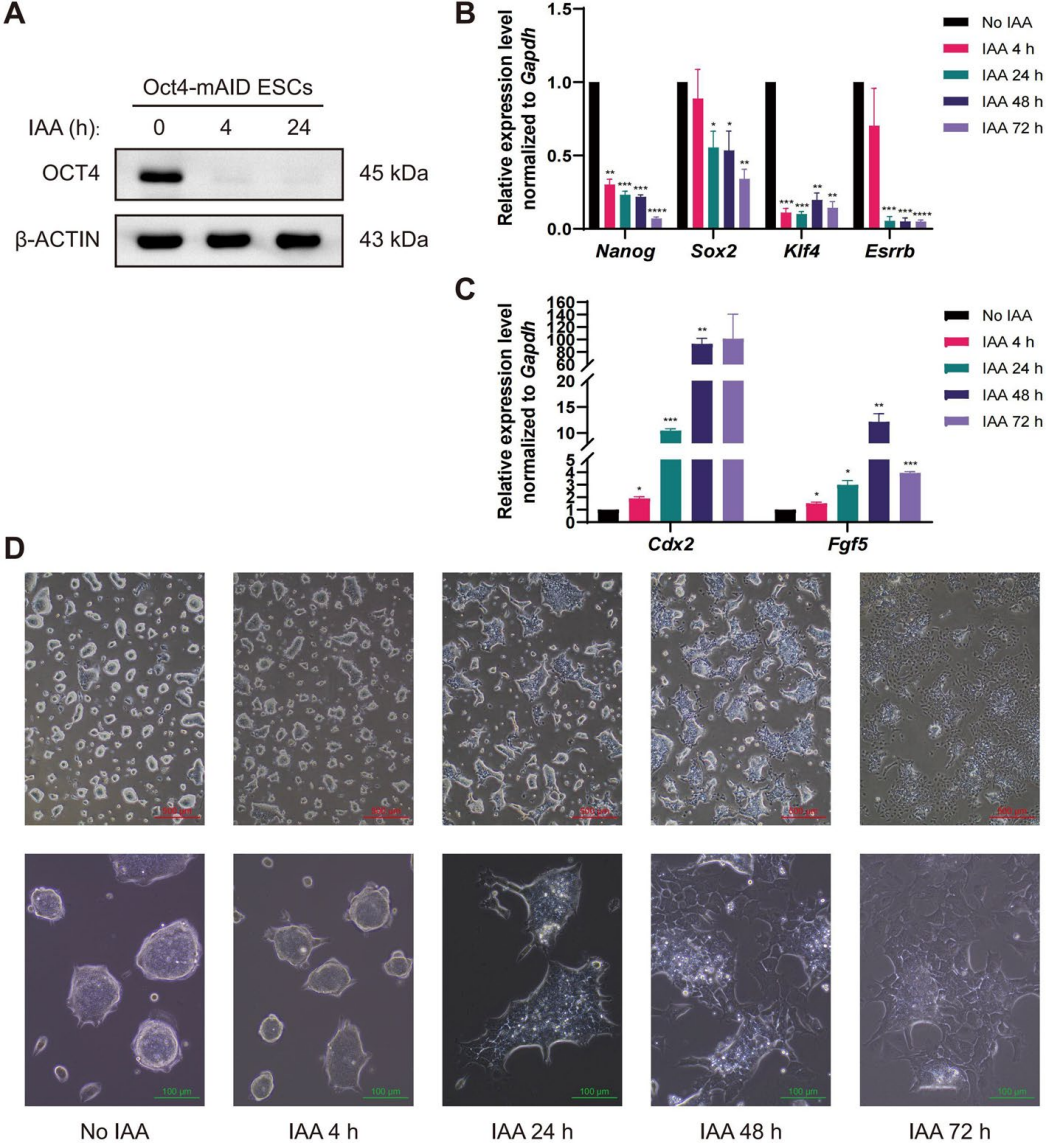
Functional verifiation of Oct4-mAID ESCs. **A** Rapid degradation of OCT4-mAID fusion protein. Western blot experiment was used to detect the degradation of OCT4-mAID fusion protein in cells treated with IAA for 0, 4 and 24 h. The levels of OCT4-mAID protein were analyzed by western blot using OCT4 antibody. β-ACTIN protein was used as a loading control. **B** Transcriptional levels of pluripotency-related genes (*Nanog*, *Sox2*, *Klf4* and *Esrrb*) were measured by qPCR in Oct4-mAID ESCs treated with or without IAA. Statistical analysis was performed using *t* test, *P < 0.05, **P < 0.01, ***P < 0.001, ****P < 0.0001. **C** The transcriptional levels of differentiation-related genes (*Cdx2*, *Fgf5*) were analyzed by qPCR in Oct4-mAID ESCs treated with or without IAA. Statistical analysis was performed using *t* test, *P < 0.05, **P < 0.01, ***P < 0.001. **D** Representative morphology of Oct4-mAID ESCs treated with or without IAA. Scale bars: 500 μm (upper panel) and 100 μm (bottom panel)

To validate the functionality of the Oct4-mAID ESCs, we first assessed the rapid degradation of the OCT4 protein following auxin induction. Western blot analysis confirmed a substantial reduction in OCT4 protein levels in the Oct4-mAID ESCs after treatment with 1 μg/mL doxycycline (Dox) and 500 μM of IAA for 4 and 24 h, respectively (Li et al. 2022) (Fig. 1A), indicating effective and rapid degradation of the mAID-tagged OCT4 protein.

Given the essential role of Oct4 in maintaining pluripotency and self-renewal in ESCs, its depletion is known to trigger differentiation (Christophersen and Helin 2010; Niwa 2001; Niwa et al. 2002; Niwa et al. 2000; Zafarana et al. 2009). Consistent with this, IAA treatment for 4 h resulted in a marked downregulation of the pluripotency-associated genes *Nanog* and *Klf4* in the Oct4-mAID ESCs (Fig. 1B), indicating the initiation of ESC differentiation. Prolonged IAA exposure for 24, 48 and 72 h further decreased the expression levels of key pluripotency markers, including *Nanog*, *Sox2*, *Klf4* and *Esrrb* (Fig. 1B). In parallel, the expression of lineage-specific markers, such as the trophectoderm-associated gene *Cdx2* and the primitive ectoderm marker *Fgf5*, increased significantly (Fig. 1C), aligning with the known repressive role of Oct4 on *Cdx2* and *Fgf5* expression (Hammachi et al. 2012; Niwa et al. 2005).

Morphological changes further corroborated the initiation of differentiation. After 4 h of IAA treatment, the Oct4-mAID ESCs exhibited a flattened morphology compared to the untreated controls (Fig. 1D). Extended treatment for 24 to 72 h led to pronounced differentiation, with cell morphology progressively resembling that of trophoblast-like cells, consistent with previous observations upon OCT4 depletion (Niwa et al. 2005). Notably, a trophoblast-like morphology was prominent after 72 h of OCT4 degradation (Fig. 1D).

Overall, our results demonstrate that rapid degradation of OCT4 in Oct4-mAID ESCs leads to significant downregulation of key pluripotency genes and upregulation of the trophoblast marker *Cdx2*, in agreement with established roles of Oct4 in pluripotency maintenance and differentiation.

### Transcriptional targets analysis of Oct4 in Oct4-mAID ESCs

**Fig. 2:**
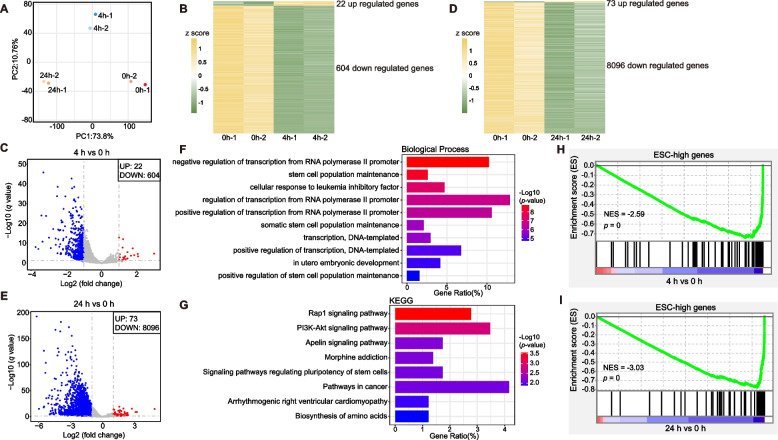
Transcriptional targets analysis of Oct4 in Oct4-mAID ESCs. **A** Principal Component Analysis (PCA) of Oct4-mAID ESCs nascent RNA-seq data treated with IAA for 0, 4 and 24 h. **B** Heat map analysis of global gene expression levels in cells treated with IAA for 0 and 4 h (fold change > 2, *P*-adj < 0.05). **C** Volcano plot analysis of global gene expression levels in cells treated with IAA for 4 h (fold change > 2, *P*-adj < 0.05). **D** Heat map analysis of global gene expression levels in cells treated with IAA for 0 and 24 h (fold change > 2, *P*-adj < 0.05). **E** Volcano plot analysis of global gene expression levels in cells treated with IAA for 24 h (fold change > 2, *P*-adj < 0.05). **F** Gene Ontology (GO) enrichment analysis of down-regulated genes at 4 h. **G** Kyoto Encyclopaedia of Genes and Genomes (KEGG) pathway enrichment analysis of down-regulated genes at 4 h. **H** Gene Set Enrichment Analysis (GSEA) of ESC-high genes in nascent RNA-seq after rapid degradation of OCT4 protein (4 h versus 0 h, FDR = 0). **I** GSEA of ESC-high genes in nascent RNA-seq after rapid degradation of OCT4 protein (24 h versus 0 h, FDR = 0)

The rapid degradation of AID-tagged transcription factors (TFs) provides a powerful method for identifying direct transcriptional targets (Atkins et al. 2020; Li et al. 2022; Ng et al. 2019; Nora et al. 2017). Nascent RNA sequencing enables the detection of immediate regulatory changes in response to various biological cues, such as developmental, environmental, disease, and metabolic signals (Wissink et al. 2019). To identify novel OCT4 target genes, we performed nascent RNA-seq on Oct4-mAID ESCs treated with IAA for 0, 4 and 24 h. Principal component analysis (PCA) revealed distinct clustering of untreated cells (0 h) compared to IAA-treated cells at both 4 and 24 h, demonstrating consistent and reproducible nascent RNA-seq data across biological replicates at each time point (Fig. 2A).

Differential gene expression analysis revealed a significant shift in gene expression following OCT4 degradation. After 4 h of IAA treatment, 22 genes were upregulated and 604 genes were downregulated (Fig. 2B-C). This trend became more pronounced after 24 h, with 73 upregulated and 8,096 downregulated genes identified (Fig. 2D-E), indicating that OCT4 depletion primarily led to a widespread reduction in gene expression in Oct4-mAID ESCs.

To investigate the functional significance of the downregulated genes, we conducted GO and KEGG pathway enrichment analyses on the downregulated genes after 4 h of IAA treatment. GO analysis showed significant enrichment for processes related to stem cell population maintenance and the cellular response to leukemia inhibitory factor (Fig. 2F). KEGG pathway analysis indicated that the downregulated genes were primarily associated with the Rap1 signaling pathway, the PI3K-Akt signaling pathway, and pathways regulating stem cell pluripotency (Fig. 2G), suggesting a critical role for these pathways in OCT4-mediated gene regulation.

Further analysis integrated data from 94 genes highly expressed in ESCs ("ESC-high genes", (Bi et al. 2019)) with the downregulated genes identified at 4- and 24- hours post-IAA treatment. GSEA demonstrated a significant downregulation of these highly expressed ESC genes following OCT4 degradation (Fig. 2H-I). This downregulation supports the notion that OCT4 is essential for sustaining the transcriptional network that maintains stem cell pluripotency.

Overall, our findings indicate that the rapid degradation of OCT4 in Oct4-mAID ESCs leads to the downregulation of genes critical for pluripotency, corroborating previous studies on the role of Oct4 in maintaining the undifferentiated state of ESCs (Boyer et al. 2005; Chambers and Tomlinson 2009; Christophersen and Helin 2010; Loh et al. 2006; Niwa 2001; Ye et al. 2020).

### Non-coding RNA analysis upon OCT4 degradation in Oct4-mAID ESCs

Non-coding RNAs play significant roles in the regulation of gene expression and cell fate (Arnold et al. 2019; Feschotte 2008; Frye et al. 2018; Li et al. 2016). The binding of OCT4 to enhancers plays a crucial role in maintaining the pluripotency of ESCs (Hnisz et al. 2013; Whyte et al. 2013; Xiong et al. 2022). Upon OCT4 depletion, there is an immediate loss of its binding to enhancer elements, impacting gene regulation (Xiong et al. 2022). To investigate the effects of OCT4 degradation on non-coding RNAs, we analyzed the expression of super-enhancers and typical enhancers, as well as their target genes, transfer RNAs (tRNAs) and transposable elements in Oct4-mAID ESCs following IAA-induced OCT4 degradation.

**Fig. 3:**
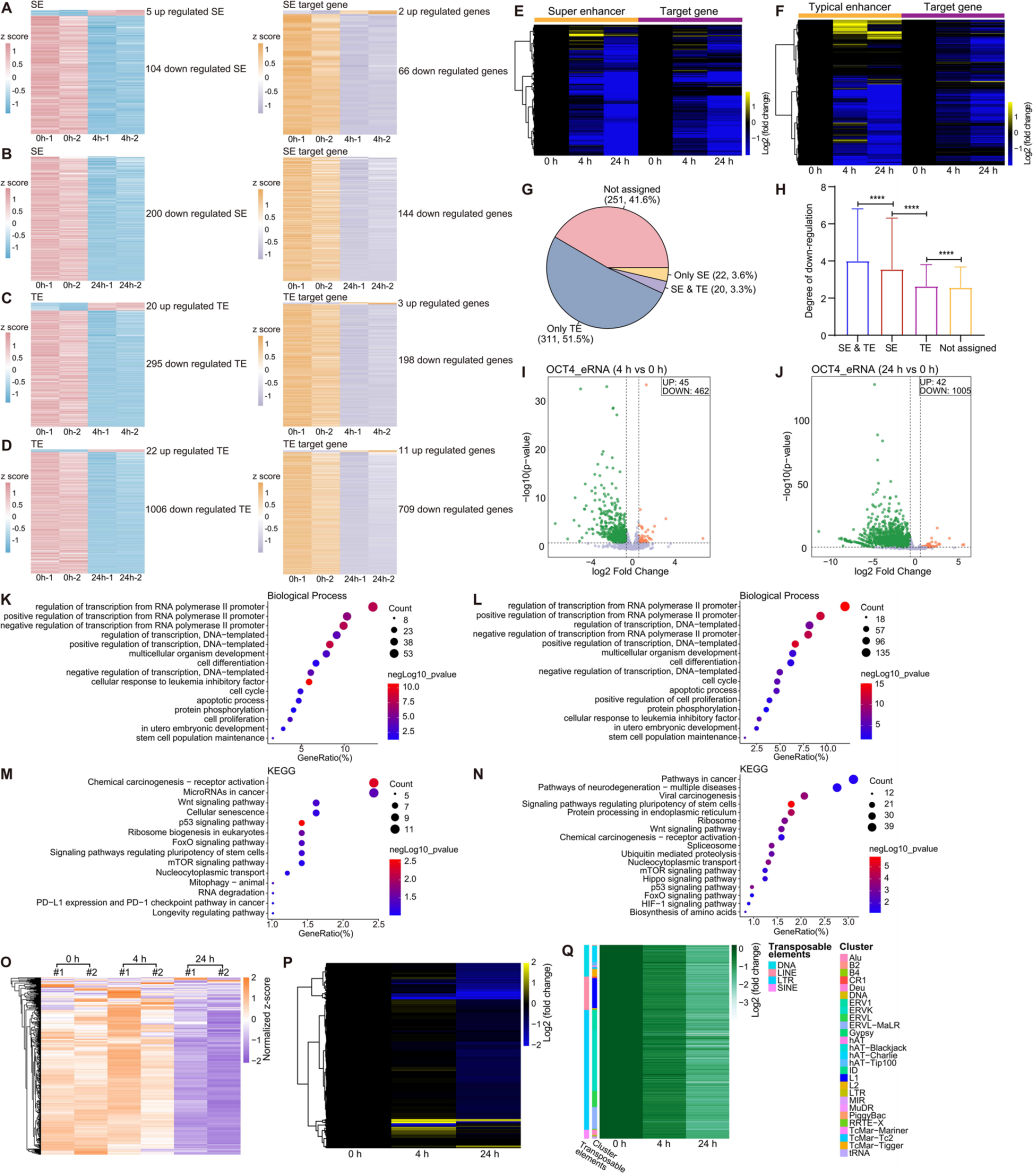
Non-coding RNA analysis upon OCT4 degradation in Oct4-mAID ESCs. **A-B** Heat maps of super-enhancer-associated transcripts (Whyte et al. 2013) (left panel) and their target genes (right panel) after 4 h (**A**) and 24 h (**B**) of IAA treatment (fold change > 1.5, *P* < 0.05). **C** Heat maps of all super-enhancers (*n* = 231) and their target genes showing fold change relative to 0 h after 4 h and 24 h of IAA treatment. **D-E** Heat maps of typical enhancer-associated transcripts (Whyte et al. 2013) (left panel) and their target genes (right panel) after 4 h (**D**) and 24 h (**E**) of IAA treatment (fold change > 1.5, *P* < 0.05). **F** Heat map analysis of all typical enhancers (*n* = 8,563) and their target genes showing fold change relative to 0 h after 4 h and 24 h of IAA treatment. **G** The proportion of 4 h down-regulated genes (*n* = 604) occupied by enhancers in different cases. SE: super-enhancers. TE: typical enhancers. "Only SE": genes occupied only by SEs. "Only TE": genes occupied only by TEs. "SE & TE": genes occupied by both SEs and TEs. "Not assigned": genes were not occupied by any enhancer. **H** The corresponding down-regulation degree of 4 h down-regulated genes (*n* = 604) occupied by enhancers in different cases. Statistical analysis was performed using *Wilcoxon* test, ****P < 0.0001. SE: super-enhancers. TE: typical enhancers. **I** Volcano plot analysis of eRNA expression levels treated with IAA for 4 h (fold change > 1.5, *P* < 0.05). **J** Volcano plot analysis of eRNA expression levels treated with IAA for 24 h (fold change > 1.5, *P* < 0.05). **K** Gene Ontology (GO) enrichment analysis of target genes corresponding to 4 h down-regulated eRNAs. **L** Gene Ontology (GO) enrichment analysis of target genes corresponding to 24 h down-regulated eRNAs. **M** Kyoto Encyclopaedia of Genes and Genomes (KEGG) pathway enrichment analysis of target genes corresponding to 4 h down-regulated eRNAs. **N** Kyoto Encyclopaedia of Genes and Genomes (KEGG) pathway enrichment analysis of target genes corresponding to 24 h down-regulated eRNAs. **O** Heat map analysis of tRNA expression levels. **P** Heat map analysis of tRNA fold change relative to 0 h. **Q** Heat map analysis of the fold changes in transposable elements expression following OCT4 degradation at the indicated time points (fold change > 1.5, *P*-adj < 0.05)

Super-enhancers are essential for regulating pluripotency in ESCs (Ma et al. 2024; Zhang et al. 2022a). The core pluripotency transcription factors Oct4, Nanog and Sox2 maintain ESC identity by binding to enhancer regions and recruiting mediator proteins (Whyte et al. 2013; Xiong et al. 2022). To assess the impact of rapid OCT4 degradation, we examined the expression levels of super-enhancers and their associated target genes after 4- and 24-h of IAA treatment (Whyte et al. 2013)(Fig. 3A-B). Following 4 h of IAA exposure, 104 (45.0%) out of 231 super-enhancers exhibited downregulated expression, while only 5 showed upregulation (Fig. 3A, left panel). Correspondingly, among the target genes of these super-enhancers, 66 were downregulated, while only 2 were upregulated (Fig. 3A, right panel). The trend intensified at 24 h, with 200 (86.6%) super-enhancers and 144 target genes downregulated, and no upregulation detected (Fig. 3B).

Further analysis revealed a substantial decrease in the expression of 231 super-enhancers and their target genes at both 4- and 24-h post-IAA treatment relative to untreated controls (Fig. 3C). Consistent with our nascent RNA-seq results (Fig. 2B-E), more super-enhancers and their target genes were significantly downregulated, with the extent of downregulation being more pronounced at 24 h (Fig. 3A-C). Notably, the downregulation of super-enhancers was more significant than that of their target genes, underscoring the profound regulatory role of super-enhancers in maintaining gene expression in ESCs.

We also examined the expression changes in typical enhancers and their target genes upon IAA-induced OCT4 degradation (Whyte et al. 2013)(Fig. 3D-E). After 4 h of IAA treatment, 295 (3.4%) typical enhancers were downregulated, while 20 were upregulated (Fig. 3D, left panel). Among the target genes, 198 exhibited downregulation, whereas 3 showed upregulation (Fig. 3D, right panel). Following 24 h of IAA exposure, 1,006 (11.7%) typical enhancers and 709 target genes were downregulated, while 22 enhancers and 11 target genes were upregulated (Fig. 3E). Similar to the trends observed for super-enhancers, more typical enhancers and their associated target genes were downregulated compared to upregulated after OCT4 degradation (Fig. 3D-F).

To further explore the regulatory relationships, we investigated enhancer-promoter (E-P) loop mechanisms, whereby enhancers regulate target gene expression (Deng et al. 2012; Mora et al. 2016). We integrated the nascent RNA differential expression data and analyzed the downregulation patterns of genes associated with super-enhancers (SEs) and typical enhancers (TEs) after 4 h of IAA treatment. Among the 604 downregulated genes that met the criteria of fold change > 2 and adjusted *P*-value (*P*-adj) < 0.05, 20 (3.3%) genes simultaneously regulated by both SEs and TEs (Fig. 3G, "SE & TE"), 22 (3.6%) regulated only by SEs (Fig. 3G, "Only SE"), and 311 (51.5%) regulated only by TEs (Fig. 3G, "Only TE"). Additionally, 251 (41.6%) downregulated genes were not linked to any enhancer (Fig. 3G, "Not assigned"). Genes co-regulated by both SEs and TEs exhibited the most significant downregulation, followed by those regulated solely by SEs, while genes regulated by TEs alone or not linked to any enhancer showed milder downregulation (Fig. 3H).

Enhancer RNAs (eRNAs) have been reported to play roles in regulating target gene expression (Arnold et al. 2019; Li et al. 2016). Therefore, we designated eRNAs and inferred putative target genes, as detailed in Table S1. Differential expression analysis revealed a significant shift in eRNA expression following OCT4 degradation. After 4 h of IAA treatment, 45 eRNAs were upregulated and 462 eRNAs were downregulated (Fig. 3I). This trend became more pronounced after 24 h of IAA induced OCT4 degradation, with 42 upregulated and 1,005 downregulated eRNAs identified (Fig. 3J), indicating that OCT4 depletion primarily led to a widespread reduction in eRNA expression in Oct4-mAID ESCs. Meanwhile, to investigate the functional significance of target genes corresponding to down-regulated eRNAs (fold change > 1.5, *P* < 0.05) after 4 and 24 h of IAA treatment, we conducted GO and KEGG pathway enrichment analyses on these target genes. GO analysis showed significant enrichment for processes related to regulation of transcription, cell differentiation, cellular response to leukemia inhibitory factor, cell cycle, apoptotic, protein phosphorylation, cell proliferation and stem cell population maintenance (Fig. 3K-L). KEGG pathway analysis indicated that these genes are involved in signaling pathways regulating pluripotency of stem cells, pathways in cancer, as well as key signaling pathways, including p53, Wnt, mTOR, FoxO, Hippo and HIF-1 signaling pathway (Fig. 3M-N).

In addition, we analyzed the expression of tRNA following OCT4 degradation (Fig. 3O-P). Minor transcriptional changes were observed at 4 h post-IAA treatment, but a global downregulation of tRNA expression became evident after 24 h, potentially reflecting a decreased protein synthesis rate associated with differentiation (Corsini et al. 2018; You et al. 2015).

Transposon-derived lncRNAs have been implicated in regulating pluripotency transitions in ESCs (Meng et al. 2023). To further investigate the role of OCT4 in this context, we analyzed the expression profiles of transposable elements following OCT4 degradation (Fig. 3Q). Differentially expressed transposable elements were identified using a threshold of fold change > 1.5 and an adjusted *P*-value (*P*-adj) < 0.05. To provide a comprehensive overview, we integrated data from both the 4-h and 24-h time points and visualized the results in a heatmap. This analysis revealed a widespread downregulation of transposable elements as early as 4 h after IAA treatment, with further suppression observed at 24 h (Fig. 3Q). These findings suggest that OCT4 may contribute to the regulation of pluripotency by modulating transposable element expression dynamics.

### Identification of direct OCT4 targets through integration of OCT4 ChIP-seq and nascent RNA-seq data

**Fig. 4:**
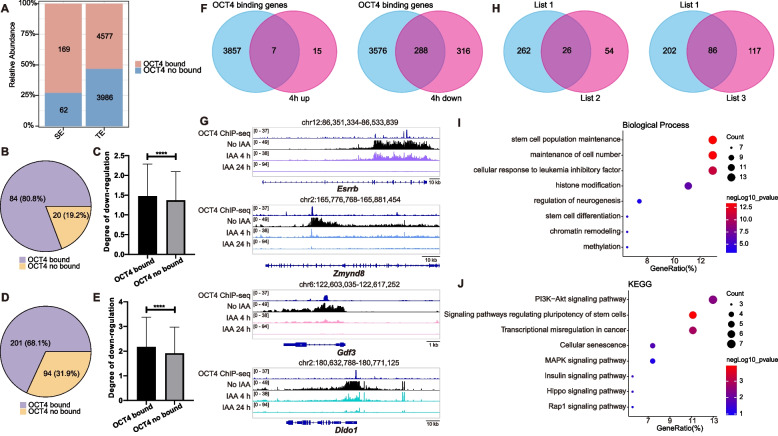
Identification of direct OCT4 targets through integration of OCT4 ChIP-seq and nascent RNA-seq data. **A** Percent stacted column chart of super-enhancers (*n* = 231) and typical enhancers (*n* = 8,563) occupied by OCT4. SE: super-enhancers. TE: typical enhancers. **B** The proportion of super-enhancers with significant transcriptional down-regulation at 4 h (fold change > 1.5, *P* < 0.05) bound or no bound by OCT4. **C** The corresponding down-regulation degree of super-enhancers with significant transcriptional down-regulation at 4 h bound or no bound by OCT4. Statistical analysis was performed using *Wilcoxon* test, ****P < 0.0001. **D** The proportion of typical enhancers with significant transcriptional down-regulation at 4 h (fold change > 1.5, *P* < 0.05) bound or no bound by OCT4. **E** The corresponding down-regulation degree of typical enhancers with significant transcriptional down-regulation at 4 h bound or no bound by OCT4. Statistical analysis was performed using *Wilcoxon* test, ****P < 0.0001. **F** Overlap analysis between OCT4 ChIP-seq peaks (GEO accession: GSM2341286) and genes significantly upregulated (left panel) and downregulated (right panel) (fold change > 2, *P*-adj < 0.05) following 4-h IAA treatment. **G** Integrative Genomic Viewer (IGV) snapshot of OCT4 ChIP-seq and nascent RNA-seq at *Esrrb*, *Zmynd8*, *Gdf3* and *Dido1* gene locus after IAA-induced OCT4 degradation (0 h, 4 h, 24 h). **H** Overlap analysis of List 1 and List 2 (left panel), List 1 and List 3 (right panel). List 1: OCT4-bound genes down-regulated at 4 h (fold change > 2, *P*-adj < 0.05), namely 288 overlapping genes in Fig. 4F. List 2: Target genes of OCT4-bound super-enhancers that down-regulated at 4 h (fold change > 1.5, *P* < 0.05). List 3: Target genes of OCT4-bound typical enhancers that down-regulated at 4 h (fold change > 1.5, *P* < 0.05). **I** Gene Ontology (GO) enrichment analysis of all genes contained in the two overlaps in (**H**). **J** Kyoto Encyclopaedia of Genes and Genomes (KEGG) pathway enrichment analysis of all genes contained in the two overlaps in (**H**)

To elucidate the regulatory impact of OCT4 binding on enhancer activity, we integrated OCT4 ChIP-seq data (GEO accession: GSM2341286) with enhancer annotations specific to mouse ESCs. Among the 231 identified super-enhancers and 8,563 typical enhancers (Whyte et al. 2013), OCT4 occupancy was detected at 169 (73.2%) super-enhancers and 4,577 (53.5%) typical enhancers (Fig. 4A), indicating a strong association between OCT4 binding and enhancer regulation.

Next, we evaluated the impact of OCT4 degradation on enhancer activity by assessing the proportion of OCT4-bound enhancers that displayed significant expression changes after 4 h of IAA treatment. Using the *Wilcoxon* test, we compared the downregulation levels of OCT4-bound versus unbound enhancers. Among the 104 super-enhancers with more than a 1.5-fold reduction in expression (Fig. 3A, left panel), 84 (80.8%) were bound by OCT4 (Fig. 4B), and the downregulation was significantly more pronounced compared to unbound super-enhancers (Fig. 4C). Similarly, of the 295 typical enhancers with over a 1.5-fold reduction in expression (Fig. 3D, left panel), 201 (68.1%) were occupied by OCT4 (Fig. 4D), and these OCT4-bound enhancers exhibited greater downregulation than the unbound ones (Fig. 4E). These findings underscore the direct role of OCT4 in regulating enhancer activity.

To identify direct target genes of OCT4, we conducted an intersection analysis between genes differentially expressed after 4 h of IAA treatment and genes associated with OCT4 binding. This analysis revealed that 7 out of 22 upregulated genes (31.8%) were directly bound by OCT4, including *Camk2n1*, *Gadd45g*, *Glrx*, *Jun*, *Kitl*, *Tfap2c* and *Wnt7b* (Fig. 4F, left panel; Table S2). Furthermore, a considerable fraction of the 604 downregulated genes—specifically, 288 genes such as *Esrrb*, *Zmynd8*, *Gdf3* and *Dido1*—also exhibited OCT4 binding at their genomic loci (Fig. 4F, right panel, G; Table S2). These findings suggest a significant concordance between OCT4 occupancy and transcriptional repression following acute OCT4 depletion.

To further refine the identification of direct OCT4 target genes, we conducted a more stringent intersection analysis. We identified the overlap between OCT4-bound genes downregulated at 4 h and target genes associated with super-enhancers, these super-enhancers were also bound by OCT4 and downregulated at 4 h. This analysis yielded 26 overlapping genes (Fig. 4H, left panel). Similarly, we intersected the downregulated OCT4-bound genes with target genes linked to OCT4-bound typical enhancers that exhibited downregulation, identifying 86 overlapping genes (Fig. 4H, right panel). By combining these two sets of results, we identified a total of 111 genes as putative or potential direct targets of OCT4.

Functional annotation of these 111 putative or potential direct OCT4 target genes using GO analysis revealed significant enrichment in biological processes such as stem cell population maintenance, stem cell differentiation, chromatin remodeling, regulation of neurogenesis, and DNA methylation (Fig. 4I). Furthermore, KEGG pathway analysis showed that these genes are involved in pathways regulating stem cell pluripotency, as well as key signaling pathways, including PI3K-Akt, Hippo, MAPK and Rap1 signaling pathway (Fig. 4J). These results align with the established role of OCT4 in ESC maintenance and differentiation, providing further insights into the molecular mechanisms underlying pluripotency and lineage specification (Boyer et al. 2005; Chambers and Tomlinson 2009; Christophersen and Helin 2010; Loh et al. 2006; Niwa 2001; Ye et al. 2020).

### OCT4 target genes identified in this study

Through our integrative analysis, we identified 288 OCT4-bound genes that were downregulated following 4 h of IAA treatment, indicating a significant transcriptional response to OCT4 degradation. Additionally, we found 22 genes that were upregulated at 4 h and 73 genes that were upregulated at 24 h post-IAA treatment, as detailed in Table S2.

Among the downregulated OCT4-bound genes, we confirmed several previously reported OCT4 targets that play essential roles in maintaining ESC pluripotency, including *Nanog* (Kuroda et al. 2005; Rodda et al. 2005), *Esrrb* (Loh et al. 2006), *Klf2* (Kotkamp et al. 2014), *Klf4* (Kotkamp et al. 2014), *Klf5* (Yan et al. 2016), *L1td1* (Närvä et al. 2012), *Phc1* (Chen et al. 2021) and *Zfp42* (Ben-Shushan et al. 1998; Hosler et al. 1993) (Table S3). These genes are known to be critical regulators of stem cell identity and underscore OCT4’s pivotal function in sustaining the ESC state. Furthermore, genes involved in histone modification, such as *Kat6b* (Cosentino et al. 2019), *Kdm3a* (Loh et al. 2007), *Kdm4c* (Loh et al. 2007) and *Rif1* (Li et al. 2015; Loh et al. 2006), were identified as OCT4 targets, highlighting the influence of OCT4 on epigenetic regulation. In the context of cell cycle control and apoptosis, *Dido1* (Liu et al. 2014) and *Gadd45g* (Jung et al. 2010; Sharov et al. 2008) were also found to be putative or potential direct targets, suggesting OCT4’s involvement in maintaining cellular proliferation and survival in ESCs.

In addition to these known targets, we identified key regulators that further support the robustness of our analysis. For example, *Tet2* (Wu et al. 2013), which functions as a DNA demethylase, was found among the downregulated OCT4-bound genes, indicating a potential link between OCT4 activity and DNA methylation dynamics. Similarly, *Fbxo15* (Tokuzawa et al. 2003), an E3 ubiquitin-protein ligase, and *Rest* (Campbell et al. 2007; Loh et al. 2006), a transcriptional repressor associated with neurogenesis regulation, were also recognized as putative or potential direct OCT4 targets, expanding the functional scope of OCT4’s regulatory network (Table S3).

Notably, our analysis revealed a set of previously unreported putative OCT4 target genes (Table S3). These genes exhibited significant transcriptional alterations upon OCT4 degradation, suggesting that their expression is directly regulated by OCT4. To validate these observations, we performed qPCR analyses of selected differentially expressed genes following 4 h and 24 h of IAA treatment. In agreement with the nascent RNA-seq data (Table S3), *Asns*, *Cdyl*, *Cdyl2*, *Cobl*, *Fhod3*, *Pcsk6*, *Pigl*, *Ssr2* and *Tenm4* were significantly downregulated at both time points (Fig. S1A-B; Table S3). Conversely, *Gadd45g*, *Glrx*, *Kitl* and *Wnt7b* were markedly upregulated at both 4 h and 24 h (Fig. S1C-D; Table S3). The roles of these newly identified OCT4-regulated genes in ESC biology remain to be determined and may provide further insight into the transcriptional network governing pluripotency and lineage commitment.

### Comparative transcriptomic analysis of OCT4 loss using AID-nascent RNA-seq and Cre/loxP approaches

To further illustrate the distinct advantages of our approach, we compared our dataset with previously reported bulk RNA-seq data from *Oct4*^*f/−*^ CreER ESCs (GEO accession: GSE168577) (Bates et al. 2021). In that study, the expression of key pluripotency-associated genes, including *Nanog*, *Esrrb*, *Tbx3*, *Klf4* and *Sall4*, did not exhibit significant downregulation following *Oct4* depletion for either 12 or 24 h. Consistently, genes associated with differentiation also showed no marked upregulation (Fig. S2A-B). In contrast, our approach using IAA-induced degradation of OCT4 protein led to a rapid and pronounced downregulation of pluripotency genes such as *Nanog*, *Esrrb*, *Klf4*, *Tfcp2l1* and *Tbx3* at *both* 4 and 24 h. Concurrently, differentiation-associated genes including *Gadd45g*, *Gadd45b*, *Nes*, *Sox11* and *Gata3* were significantly upregulated (Fig. S2C-D). Collectively, these observations highlight distinct transcriptional dynamics resulting from gradual versus acute OCT4 protein depletion.

To further investigate the molecular consequences of these approaches, we performed an overlap analysis of differentially expressed genes identified in *Oct4*^*f/−*^ ESCs and Oct4-mAID ESCs (Fig. S2E-F; Table S4). We first intersected the genes downregulated in *Oct4*^*f/−*^ ESCs following 24 h of 4-OHT treatment with those downregulated in Oct4-mAID ESCs after 4 h of IAA treatment. In the *Oct4*^*f/−*^ system, only 28 genes were significantly downregulated at 24 h, whereas the Oct4-mAID system revealed 604 downregulated genes at 4 h and 8,096 at 24 h (Fig. S2E-F; Table S4), highlighting the superior sensitivity and temporal resolution afforded by our degron-based strategy. Among the genes downregulated in *Oct4*^*f/−*^ ESCs following 24 h of 4-OHT treatment, 7 overlapped with those downregulated in Oct4-mAID ESCs after 4 h of IAA treatment (Fig. S2E), and 13 overlapped with those downregulated after 24 h of IAA treatment (Fig. S2F). Notably, key pluripotency regulators such as *Nr0b1* and *Foxd3* were present in both overlapping sets (Table S4). The limited overlap between the datasets indicates that our approach captures a more comprehensive and temporally dynamic repertoire of OCT4-dependent target genes, including novel candidates that may be overlooked by conventional Cre/loxP-based strategies.

Collectively, these results validate the robustness of our approach and support its broader applicability for dissecting transcriptional regulatory networks beyond OCT4.

## Discussion

Oct4 is one of the most critical transcription factors for maintaining the pluripotency of ESCs. Understanding the regulation of its target genes is vital for comprehending how pluripotency is maintained and how differentiation is regulated. Previous studies primarily identified OCT4 target genes using methods such as gene knockout or knockdown (Elbashir et al. 2001; Fire et al. 1998; Sauer and Henderson, 1988). However, these approaches have limitations, including off-target effects and incomplete degradation of target proteins (El-Brolosy and Stainier 2017; Housden et al. 2017; Jackson et al. 2003; Wang et al. 2020). Moreover, protein degradation is often time-consuming, which may allow cells sufficient time to establish compensatory mechanisms or adaptive responses, making it challenging to accurately interpret direct phenotypic changes following the loss of protein function (El-Brolosy and Stainier 2017; Housden et al. 2017; Sacher et al. 2008; Weiss et al. 2007; Wood et al. 2016). This can impact the precise study of the functions of proteins involved in highly dynamic biological processes such as the cell cycle, neural activity, and ESC differentiation (Atkins et al. 2020; Ng et al. 2019; Nora et al. 2017).

The auxin-inducible degron (AID) system enables rapid and reversible degradation of target proteins, thereby preventing interference from secondary effects (Weiss et al. 2007; Wood et al. 2016). This system is particularly advantageous for accurately analyzing phenotypes resulting from the loss of protein function (Natsume et al. 2016; Nishimura et al. 2009). To date, the AID system has been successfully applied to cell lines derived from vertebrates, fruit flies, nematodes, budding yeast, chickens, mice, hamsters, monkeys, and humans (Holland et al. 2012; Morawska and Ulrich 2013; Nishimura et al. 2009; Nishimura et al. 2020; Nora et al. 2017; Trost et al. 2016; Zhang et al. 2015), making it a powerful tool for exploring new functions of key pluripotency genes in ESCs (Atkins et al. 2020; Ng et al. 2019; Nora et al. 2017). For example, in recent years, researchers applied the AID system in mouse ESCs to achieve rapid degradation of the transcription factor CTCF. Using high-throughput sequencing techniques such as Hi-C and ChIP-seq, they demonstrated CTCF’s significant guiding role in chromatin folding, providing new insights into the molecular mechanisms of mammalian chromatin architecture (Nora et al. 2017).

Traditional RNA-seq is used to measure mRNA expression levels under steady-state conditions (Bates et al. 2021; Festuccia et al. 2012). However, gene expression in living organisms is fundamentally a highly dynamic process. Conventional RNA-seq cannot detect subtle and rapid changes during complex transcriptional responses, nor can it identify unstable non-coding RNAs (such as enhancer RNAs). In contrast, emerging nascent RNA-seq technologies can quantitatively assess newly transcribed RNA, thereby enabling studies of RNA dynamics that are critically important. By utilizing various nascent RNA-seq technologies, researchers have demonstrated that nascent RNA directly regulates transcription and indicated that the specificity of nascent RNA in structure and sequence can affect transcriptional elongation, pausing, and termination, as well as the binding of chromatin modifiers and enhancer RNAs, thereby deepening our understanding of RNA dynamics (Skalska et al. 2017).

This study systematically analyzes the nascent RNAs regulated during the rapid degradation of OCT4 in an Oct4-mAID cell line, utilizing 5-ethynyl uridine (EU)-based nascent RNA capture techniques. This approach offers a new perspective on the transcriptional regulatory network mediated by the master transcription factor Oct4. By integrating the AID system with nascent RNA-seq technologies, we identified several previously unreported potential new targets of Oct4 (Table S3). Notably, *Cobl*, a novel actin nucleating factor, dynamically influences neuronal morphology by regulating actin (Ahuja et al. 2007); *Pigl* encodes a key enzyme in the GPI-anchor biosynthesis pathway, which is involved in the attachment of GPI-anchor to target proteins (Álvarez-Sánchez et al. 2024); and *Ssr2*, a critical gene involved in protein folding and processing within the endoplasmic reticulum, functions as an oncogene in hepatocellular carcinoma (Chen et al. 2022), all exhibited over fourfold reduction in transcriptional expression following 4 h of OCT4 degradation.

Additionally, we observed multiple genes that were upregulated more than twofold after four hours of OCT4 degradation, including *Kitl* (known as stem cell factor) (Chang et al. 2020), *Glrx* (closely associated with the tumor immune microenvironment) (Chang et al. 2020), *Wnt7b* (involved in regulating the Wnt signaling pathway) (Liu et al. 2022; Lv et al. 2018), and *Camk2n1* (which has cancer-suppressing functions) (Zhang et al. 2022b). Given the limited prior reports on genes inhibited by Oct4, these results may provide interesting directions for future related research.

## Conclusions

In summary, our study provided a foundational dataset for further in-depth exploration of the direct transcriptional targets of OCT4 and the related regulatory networks. The strategies employed in this study could also be applied to transcription factor-related research in various other cellular contexts. The specific mechanisms by which Oct4 influences its targets remain to be fully elucidated and warrant further investigation.

## Methods

### Cell culture

The Oct4-mAID ESCs used in this study were generated by Li et al*.* (Li et al. 2022). Oct4-mAID ESCs were cultured as previously described (Zhang et al. 2019). Briefly, Oct4-mAID ESCs were cultured on gelatin-coated Petri dishes with ES medium (DMEM supplemented with 10% fetal bovine serum, 2 mM L-glutamine, 50 mg/mL penicillin, 80 mg/mL streptomycin, 0.1 mM 2-Mercaptoethanol (Gibco), and 10^3^ units/mL of leukemia inhibitory factor (LIF, ORIGENE)) at 37℃ and 5% CO_2_. Oct4-mAID ESCs were dissociated with 0.05% trypsin in EDTA (Gibco). The Oct4-mAID ESCs grown in the presence of 1 μg/mL of doxycycline. To induce the degradation of AID tagged proteins, Oct4-mAID ESCs were cultured in the prescence of 500 μM of indole-3-acetic acid (IAA, Sigma) was added to cells for the indicated time.

### Western blot

Proteins were separated on SDS-PAGE and transferred to PVDF membranes (Millipore). The membranes were blocked for 1 h at room temperature with 5% milk in TBST (0.1% Tween-20 in tris-buffered saline), and then incubated overnight at 4℃ with the primary antibodies. After three washes with TBST, the membranes were incubated for 2 h at room temperature with HRP-conjugated secondary antibodies. Following three additional washes with TBST, the protein signals were generated with ECL (Millipore) and detected with FluorChem E (Protein Simple). The antibody information was provided in Table S5.

### Quantitative real-time PCR (RT-qPCR)

Total RNA was isolated with TRNzol Universal RNA Reagent (TIANGEN #DP424). cDNA was synthesized with ReverTra Ace qPCR RT Kit (TOYOBO #FSQ-101). Real-time PCR was performed with PerfectStart Green qPCR SuperMix (2 ×) (TRANSGEN #AQ601-04). Quantitative RT-qPCR was performed in QuantStudio™ 3 real-time quantitative PCR instrument (Applied Biosystems) following the protocol of initial denaturation at 95℃ for 30 s, 40 cycles of 95℃ for 5 s, 60℃ for 30 s. Gene expression was determined relative to *Gapdh* transcript levels. The relative gene expression data were analyzed using the 2^−ΔΔCT^ method (Livak and Schmittgen 2001). All primers for qPCR were listed in Table S5.

### Nascent RNA-sequencing

The cells at 70% ~ 80% confluency were treated with 1 mM EU for 15 min. The EU labelling was stopped by direct addition of TRI Reagent to cells. Total RNA was isolated and treated with DNase I. EU-labelled spike-in RNA were added to each sample for calibration. The RNA was then biotinylated with click chemistry and fragmented with NaOH. After RNA fragmentation, all biotinylated RNAs were pulled down onto Dynabeads™ MyOne™ Streptavidin C1 (Invitrogen) by immunoprecipitation. The 3´-phosphate groups were removed using FastAP, the 3´-RNA-adapter was ligated using T4 RNA ligase 2 (New England Biolabs). cDNA was synthesized through reverse transcription reaction using specific RT-Primer. Dynabeads™ MyOne™ Silane (Invitrogen) were used to enrich single cDNA. T4 RNA ligase 1 (New England Biolabs) was used to ligate the 5´-DNA-adapter. The cDNA was purified for next-generation sequencing library construction.

### Nascent RNA-seq analysis

The adapter and low-quality sequences were removed from raw reads using fastp. The remaining reads were aligned to the mouse reference genome (mm10) or spike-in reference using Hisat2 with default parameters. UMI-tools was employed to identify UMIs and remove PCR duplicates. Principal component analysis (PCA) was conducted with the R package FactoMineR. Differential gene expression analysis was performed with featureCounts for read quantification using default parameters. To adjust for potential technical variations, DESeq2 was used for normalization and differential expression analysis, incorporating spike-in controls to enhance calibration accuracy. Differentially expressed genes (DEGs) were identified based on a fold change > 2 and an adjusted *P*-value (*P*-adj) < 0.05. For enhancer RNA (eRNA) analysis, primary transcripts were identified across the genome using HOMER. Transcripts overlapping protein-coding genes, antisense regions, or specific non-coding RNAs (e.g., rRNAs, snRNAs, miRNAs, snoRNAs) were excluded. Those with transcription start sites (TSS) within enhancer regions (as per EnhancerAtlas 2.0) were designated as putative enhancer RNAs. DESeq2 was applied for normalization and differential expression analysis (thresholds: fold change > 1.5, *P* < 0.05).

### Enhancer RNA (eRNA) identification

Tag directories were generated from deduplicated SAM files using the makeTagDirectory function in HOMER. De novo transcript units were identified using HOMER's findPeaks algorithm in GRO-seq mode (-style groseq). Transcription start sites (TSSs) of intergenic transcripts were intersected with annotated enhancer regions from the EnhancerAtlas 2.0 database using BEDTools, and overlapping loci were designated as eRNA candidates. For expression quantification, a custom GTF file containing merged eRNA coordinates was constructed and used as input for featureCounts. Differential expression analysis was conducted with DESeq2, applying a significance threshold of fold change > 1.5 and *P* < 0.05. Putative target genes were inferred based on genomic proximity using annotatePeaks.pl.

### GO enrichment analysis and KEGG pathway enrichment analysis

Gene Ontology (GO) enrichment analysis and Kyoto Encyclopedia of Genesand Genomes (KEGG) pathway enrichment analysis were perfommed using the DAVID (Database for Annotation, Visualization and Integrated Discovery) web site (https://david.ncifcrf.gov/).

### Gene set enrichment analysis (GSEA)

Gene set enrichment analysis (GSEA) was performed using the GSEA 4.2.3 software.

### Transposable element annotation information

The transposable element analysis was conducted using the scTE software from Jiekai Chen's research group (https://github.com/JiekaiLab/scTE) (He et al. 2021), with default parameters. The downloaded transposable elements of mm10 (http://hgdownload.soe.ucsc.edu/goldenPath/mm10/database/rmsk.txt.gz) were annotated.

### Data visualization

Bedgraphs and bigwig format were obtained for visualization in Integrative Genomics Viewer (IGV). Data were performed in RStudio and visualized using the ggplot2 library and R packages pheatmap and ComplexHeatmap.

## Supplementary Information

Supplementary Material 1. **Fig. S1** qPCR validation of putative Oct4 target genes. **A-B** Relative transcript levels of the indicated downregulated Oct4 target genes were measured by qPCR in Oct4-mAID ESCs after 4 h (**A**) and 24 h (**B**) of IAA treatment. **C-D** Transcriptional levels of the indicated upregulated target genes were measured by qPCR in Oct4-mAID ESCs after 4 h (**C**) and 24 h (**D**) of IAA treatment. Gene expression was normalized to *Gapdh* and presented relative to untreated controls. Statistical significance was assessed by two-tailed unpaired t-test; *P < 0.05, **P < 0.01, ***P < 0.001, ****P < 0.0001.

Supplementary Material 2. **Fig. S2** Comparative transcriptomic profiling following OCT4 depletion in Oct4-mAID and *Oct4*^*f/-*^ ESCs. **A-B** MA plots showing global gene expression changes in *Oct4*^*f/-*^ ESCs following 4-OHT treatment for 12 h (**A**) and 24 h (**B**) (Bates et al., 2021). **C-D** MA plots depicting transcriptomic changes in Oct4-mAID ESCs upon IAA treatment for 4 h (**C**) and 24 h (**D**). Differentially expressed genes (fold change > 2, *P*-adj < 0.05) are highlighted in green. Representative pluripotency-associated genes are marked in blue, and differentiation-associated genes in red. **E-F** Venn diagrams showing the overlap of differentially expressed genes between *Oct4*^*f/-*^ and Oct4-mAID ESCs. (**E**) Overlap of downregulated genes after 24 h 4-OHT treatment in *Oct4*^*f/-*^ ESCs and those downregulated after 4 h IAA treatment in Oct4-mAID ESCs. (**F**) Overlap of downregulated genes following 24 h treatments in both systems. (Differential expression defined as fold change > 2, *P*-adj < 0.05).

Supplementary Material 3. **Table S1**. Designated eRNAs and inferred putative target genes, related to Figure 3**I**-**J**

Supplementary Material 4. **Table S2**. OCT4 target genes identified in this study

Supplementary Material 5. **Table S3A**. Summary of Oct4 putative direct targets reported in the literature. Table S3B. Newly identified potential direct targets of Oct4 [Akerberg et al. 2022; Antao et al. 2021; Aygün et al. 2021; Berger et al. 2024; Bygrave et al. 2023; Cai et al. 2022; Chen et al. 2022a; Cooke et al. 2019; Deng et al. 2022; Desfougères et al. 2019; Gatchalian et al. 2018; He et al. 2023; Hsieh et al. 2016; Jin et al. 2020; Khan et al. 2024; Lennartsson et al. 2012; Leong et al. 2017; Liu et al. 2017; Liu et al. 2020; Markus-Koch et al. 2017; Miki and Großhans 2013; Ngubo et al. 2023; Ohbayashi et al. 2012; Prieto-Garcia et al. 2021; Rykaczewska et al. 2020; Sevilla and Grichnik 2024; Shi et al. 2022; Siouda et al. 2020; Sulistomo et al. 2019; Thomas et al.^2009^; Toledo et al. 2007; Wang et al. 2018; Wang et al. 2021; Xie et al. 2011; Yang et al. 2015; Yang et al. 2024; Yao et al. 2024; Yi et al. 2021; Yu et al. 2019; Zhao et al. 2024; Zhou et al. 2020; Zhou et al. 2020].

Supplementary Material 6. **Table S4**. Differentially expressed genes identified in *Oct4*^*f/-*^ ESCs and Oct4-mAID ESCs, related to Figure S2**E**-**F**

Supplementary Material 7. **Table S5A**. Antibody information for Western blot. Table S5B. Primers for qPCR

## Data Availability

The raw RNA-seq data were deposited to the NCBI SRA database under accession number (SRR32254951, SRR32254952, SRR32254953, SRR32254954, SRR32254955, SRR32254956, SRR32254957, SRR32254958). The data will be released to the public upon publication. All other data of this study are available from the corresponding authors upon reasonable request.

## Abbreviations

ESCs: Embryonic stem cells
ChIP-seq: Chromatin immunoprecipitation sequencing
RNAi: RNA interference
AID: Auxin-inducible degron
5-EU: 5-Ethynyl uridine
mESCs: Mouse embryonic stem cells
TADs: Topological-associated domains
KO: Knockout
RNA-seq: RNA sequencing
Dox: Doxycycline
TFs: Transcription factors
PCA: Principal component analysis
tRNAs: Transfer RNAs
E-P: Enhancer-promoter
SEs: Super-enhancers
TEs: Typical enhancers
eRNAs: Enhancer RNAs
DEGs: Diferentially expressed genes
TSS: Transcription start sites
GO: Gene Ontology
KEGG: Kyoto Encyclopedia of Genes and Genomes
GSEA: Gene set enrichment analysis
IGV: Integrative Genomics Viewer

## References

[CR1] Ahuja R, Pinyol R, Reichenbach N, Custer L, Klingensmith J, Kessels MM, et al. Cordon-bleu is an actin nucleation factor and controls neuronal morphology. Cell. 2007;131(2):337–50. 10.1016/j.cell.2007.08.030. 17956734 10.1016/j.cell.2007.08.030 PMC2507594

[CR2] Akerberg AA, Trembley M, Butty V, Schwertner A, Zhao L, Beerens M, et al. RBPMS2 is a myocardial-enriched splicing regulator required for cardiac function. Circ Res. 2022;131(12):980–1000. 10.1161/circresaha.122.321728. 36367103 10.1161/CIRCRESAHA.122.321728 PMC9770155

[CR3] Álvarez-Sánchez A, Grinat J, Doria-Borrell P, Mellado-López M, Pedrera-Alcócer É, Malenchini M, et al. The GPI-anchor biosynthesis pathway is critical for syncytiotrophoblast differentiation and placental development. Cell Mol Life Sci. 2024;81(1):246. 10.1007/s00018-024-05284-2. 38819479 10.1007/s00018-024-05284-2 PMC11143174

[CR4] Antao AM, Kaushal K, Das S, Singh V, Suresh B, Kim KS, et al. USP48 governs cell cycle progression by regulating the protein level of Aurora B. Int J Mol Sci. 2021. 10.3390/ijms22168508. 34445214 10.3390/ijms22168508 PMC8395203

[CR5] Arnold PR, Wells AD, Li XC. Diversity and emerging roles of enhancer RNA in regulation of gene expression and cell fate. Front Cell Dev Biol. 2019;7:377. 10.3389/fcell.2019.00377. 31993419 10.3389/fcell.2019.00377 PMC6971116

[CR6] Atkins A, Xu MJ, Li M, Rogers NP, Pryzhkova MV, Jordan PW. SMC5/6 is required for replication fork stability and faithful chromosome segregation during neurogenesis. Elife. 2020. 10.7554/eLife.61171. 33200984 10.7554/eLife.61171 PMC7723410

[CR7] Aygün I, Miki TS. Nuclear rNA regulation by XRN2 and XTBD Family Proteins. Cell Struct Funct. 2021;46(2):73–8. 10.1247/csf.21041. 34483148 10.1247/csf.21041 PMC10511037

[CR8] Bates LE, Alves MRP, Silva JCR. Auxin-degron system identifies immediate mechanisms of OCT4. Stem Cell Reports. 2021;16(7):1818–31. 10.1016/j.stemcr.2021.05.016. 34143975 10.1016/j.stemcr.2021.05.016 PMC8282470

[CR9] Ben-Shushan E, Thompson JR, Gudas LJ, Bergman Y. Rex-1, a gene encoding a transcription factor expressed in the early embryo, is regulated via Oct-3/4 and Oct-6 binding to an octamer site and a novel protein, Rox-1, binding to an adjacent site. Mol Cell Biol. 1998;18(4):1866–78. 10.1128/mcb.18.4.1866. 9528758 10.1128/mcb.18.4.1866 PMC121416

[CR10] Berger H, Gerstner S, Horstmann MF, Pauli S, Borchers A. Fbrsl1 is required for heart development in *Xenopus laevis* and de novo variants in FBRSL1 can cause human heart defects. Dis Model Mech. 2024. 10.1242/dmm.050507. 38501224 10.1242/dmm.050507 PMC11128277

[CR11] Bi X, Xu Y, Li T, Li X, Li W, Shao W, et al. RNA targets ribogenesis factor WDR43 to chromatin for transcription and pluripotency control. Mol Cell. 2019;75(1):102-16.e9. 10.1016/j.molcel.2019.05.007. 31128943 10.1016/j.molcel.2019.05.007

[CR12] Boyer LA, Lee TI, Cole MF, Johnstone SE, Levine SS, Zucker JP, et al. Core transcriptional regulatory circuitry in human embryonic stem cells. Cell. 2005;122(6):947–56. 10.1016/j.cell.2005.08.020. 16153702 10.1016/j.cell.2005.08.020 PMC3006442

[CR13] Bygrave AM, Sengupta A, Jackert EP, Ahmed M, Adenuga B, Nelson E, et al. Btbd11 supports cell-type-specific synaptic function. Cell Rep. 2023;42(6):112591. 10.1016/j.celrep.2023.112591. 37261953 10.1016/j.celrep.2023.112591 PMC10592477

[CR14] Cai DJ, Zhang ZY, Bu Y, Li L, Deng YZ, Sun LQ, et al. Asparagine synthetase regulates lung-cancer metastasis by stabilizing the β-catenin complex and modulating mitochondrial response. Cell Death Dis. 2022;13(6):566. 10.1038/s41419-022-05015-0. 35739087 10.1038/s41419-022-05015-0 PMC9226154

[CR15] Campbell PA, Perez-Iratxeta C, Andrade-Navarro MA, Rudnicki MA. Oct4 targets regulatory nodes to modulate stem cell function. PLoS ONE. 2007;2(6):e553. 10.1371/journal.pone.0000553. 17579724 10.1371/journal.pone.0000553 PMC1891092

[CR16] Chambers I, Tomlinson SR. The transcriptional foundation of pluripotency. Development. 2009;136(14):2311–22. 10.1242/dev.024398. 19542351 10.1242/dev.024398 PMC2729344

[CR17] Chang Y, Li G, Zhai Y, Huang L, Feng Y, Wang D, et al. Redox regulator GLRX is associated with tumor immunity in glioma. Front Immunol. 2020;11:580934. 10.3389/fimmu.2020.580934. 33329553 10.3389/fimmu.2020.580934 PMC7734322

[CR18] Chen X, Xu H, Yuan P, Fang F, Huss M, Vega VB, et al. Integration of external signaling pathways with the core transcriptional network in embryonic stem cells. Cell. 2008;133(6):1106–17. 10.1016/j.cell.2008.04.043. 18555785 10.1016/j.cell.2008.04.043

[CR19] Chen L, Tong Q, Chen X, Jiang P, Yu H, Zhao Q, et al. PHC1 maintains pluripotency by organizing genome-wide chromatin interactions of the Nanog locus. Nat Commun. 2021;12(1):2829. 10.1038/s41467-021-22871-0. 33990559 10.1038/s41467-021-22871-0 PMC8121881

[CR20] Chen M, Shan L, Gan Y, Tian L, Zhou J, Zhu E, et al. Metastasis suppressor 1 controls osteoblast differentiation and bone homeostasis through regulating Src-Wnt/β-catenin signaling. Cell Mol Life Sci. 2022;79(2):107. 10.1007/s00018-022-04147-y. 35094173 10.1007/s00018-022-04147-y PMC11072310

[CR21] Chen F, Chen, Wang J, Zhang S, Chen M, Zhang X, et al. Overexpression of SSR2 promotes proliferation of liver cancer cells and predicts prognosis of patients with hepatocellular carcinoma. J Cell Mol Med. 2022a;26(11):3169-82. 10.1111/jcmm.17314. 10.1111/jcmm.17314 PMC9170819 35481617

[CR22] Christophersen NS, Helin K. Epigenetic control of embryonic stem cell fate. J Exp Med. 2010;207(11):2287–95. 10.1084/jem.20101438. 20975044 10.1084/jem.20101438 PMC2964577

[CR23] Cooke A, Schwarzl T, Huppertz I, Kramer G, Mantas P, Alleaume AM, et al. The RNA-Binding Protein YBX3 controls amino acid levels by regulating SLC mRNA abundance. Cell Rep. 2019;27(11):3097-106.e5. 10.1016/j.celrep.2019.05.039. 31189097 10.1016/j.celrep.2019.05.039

[CR24] Corsini NS, Peer AM, Moeseneder P, Roiuk M, Burkard TR, Theussl HC, et al. Coordinated Control of mRNA and rRNA Processing Controls Embryonic Stem Cell Pluripotency and Differentiation. Cell Stem Cell. 2018;22(4):543-58.e12. 10.1016/j.stem.2018.03.002. 29625069 10.1016/j.stem.2018.03.002

[CR25] Cosentino MS, Oses C, Vázquez Echegaray C, Solari C, Waisman A, Álvarez Y, et al. Kat6b modulates Oct4 and Nanog binding to chromatin in embryonic stem cells and is required for efficient neural differentiation. J Mol Biol. 2019;431(6):1148–59. 10.1016/j.jmb.2019.02.012. 30790630 10.1016/j.jmb.2019.02.012

[CR26] Deng W, Lee J, Wang H, Miller J, Reik A, Gregory PD, et al. Controlling long-range genomic interactions at a native locus by targeted tethering of a looping factor. Cell. 2012;149(6):1233–44. 10.1016/j.cell.2012.03.051. 22682246 10.1016/j.cell.2012.03.051 PMC3372860

[CR27] Deng G, Luo Y, Zhang Y, Zhang J, He Z. Enabled homolog (ENAH) regulated by RNA binding protein splicing factor 3b subunit 4 (SF3B4) exacerbates the proliferation, invasion and migration of hepatocellular carcinoma cells via Notch signaling pathway. Bioengineered. 2022;13(2):2194–206. 10.1080/21655979.2021.2023983. 35030977 10.1080/21655979.2021.2023983 PMC8973836

[CR28] Desfougères Y, Wilson MSC, Laha D, Miller GJ, Saiardi A. ITPK1 mediates the lipid-independent synthesis of inositol phosphates controlled by metabolism. Proc Natl Acad Sci U S A. 2019;116(49):24551–61. 10.1073/pnas.1911431116. 31754032 10.1073/pnas.1911431116 PMC6900528

[CR29] Elbashir SM, Harborth J, Lendeckel W, Yalcin A, Weber K, Tuschl T. Duplexes of 21-nucleotide RNAs mediate RNA interference in cultured mammalian cells. Nature. 2001;411(6836):494–8. 10.1038/35078107. 11373684 10.1038/35078107

[CR30] El-Brolosy MA, Stainier DYR. Genetic compensation: a phenomenon in search of mechanisms. PLoS Genet. 2017;13(7):e1006780. 10.1371/journal.pgen.1006780. 28704371 10.1371/journal.pgen.1006780 PMC5509088

[CR31] Elkenani M, Mohamed BA. Murine embryonic stem cell culture, self-renewal, and differentiation. Methods Mol Biol. 2022;2520:265–73. 10.1007/7651_2021_447. 34724189 10.1007/7651_2021_447

[CR32] Feschotte C. Transposable elements and the evolution of regulatory networks. Nat Rev Genet. 2008;9(5):397–405. 10.1038/nrg2337. 18368054 10.1038/nrg2337 PMC2596197

[CR33] Festuccia N, Osorno R, Halbritter F, Karwacki-Neisius V, Navarro P, Colby D, et al. Esrrb is a direct Nanog target gene that can substitute for Nanog function in pluripotent cells. Cell Stem Cell. 2012;11(4):477–90. 10.1016/j.stem.2012.08.002. 23040477 10.1016/j.stem.2012.08.002 PMC3473361

[CR34] Fire A, Xu S, Montgomery MK, Kostas SA, Driver SE, Mello CC. Potent and specific genetic interference by double-stranded RNA in Caenorhabditis elegans. Nature. 1998;391(6669):806–11. 10.1038/35888. 9486653 10.1038/35888

[CR35] Frye M, Harada BT, Behm M, He C. RNA modifications modulate gene expression during development. Science. 2018;361(6409):1346–9. 10.1126/science.aau1646. 30262497 10.1126/science.aau1646 PMC6436390

[CR36] Gallego Romero I, Pai AA, Tung J, Gilad Y. RNA-seq: impact of RNA degradation on transcript quantification. BMC Biol. 2014;12:42. 10.1186/1741-7007-12-42. 24885439 10.1186/1741-7007-12-42 PMC4071332

[CR37] Gatchalian J, Malik S, Ho J, Lee DS, Kelso TWR, Shokhirev MN, et al. A non-canonical BRD9-containing BAF chromatin remodeling complex regulates naive pluripotency in mouse embryonic stem cells. Nat Commun. 2018;9(1):5139. 10.1038/s41467-018-07528-9. 30510198 10.1038/s41467-018-07528-9 PMC6277444

[CR38] Hammachi F, Morrison GM, Sharov AA, Livigni A, Narayan S, Papapetrou EP, et al. Transcriptional activation by Oct4 is sufficient for the maintenance and induction of pluripotency. Cell Rep. 2012;1(2):99–109. 10.1016/j.celrep.2011.12.002. 22832160 10.1016/j.celrep.2011.12.002 PMC3778438

[CR39] He J, Babarinde IA, Sun L, Xu S, Chen R, Shi J, et al. Identifying transposable element expression dynamics and heterogeneity during development at the single-cell level with a processing pipeline scTE. Nat Commun. 2021;12(1):1456. 10.1038/s41467-021-21808-x. 33674594 10.1038/s41467-021-21808-x PMC7935913

[CR40] He R, Zhang X, Wu Y, Weng Z, Li L. TTC7B is a new prognostic biomarker in head and neck squamous cell carcinoma linked to immune infiltration and ferroptosis. Cancer Med. 2023;12(24):22354–69. 10.1002/cam4.6715. 37990988 10.1002/cam4.6715 PMC10757123

[CR41] Hnisz D, Abraham BJ, Lee TI, Lau A, Saint-André V, Sigova AA, et al. Super-enhancers in the control of cell identity and disease. Cell. 2013;155(4):934–47. 10.1016/j.cell.2013.09.053. 24119843 10.1016/j.cell.2013.09.053 PMC3841062

[CR42] Holland AJ, Fachinetti D, Han JS, Cleveland DW. Inducible, reversible system for the rapid and complete degradation of proteins in mammalian cells. Proc Natl Acad Sci U S A. 2012;109(49):E3350–7. 10.1073/pnas.1216880109. 23150568 10.1073/pnas.1216880109 PMC3523849

[CR43] Hosler BA, Rogers MB, Kozak CA, Gudas LJ. An octamer motif contributes to the expression of the retinoic acid-regulated zinc finger gene Rex-1 (Zfp-42) in F9 teratocarcinoma cells. Mol Cell Biol. 1993;13(5):2919–28. 10.1128/mcb.13.5.2919-2928.1993. 8474450 10.1128/mcb.13.5.2919-2928.1993 PMC359685

[CR44] Housden BE, Muhar M, Gemberling M, Gersbach CA, Stainier DY, Seydoux G, et al. Loss-of-function genetic tools for animal models: cross-species and cross-platform differences. Nat Rev Genet. 2017;18(1):24–40. 10.1038/nrg.2016.118. 27795562 10.1038/nrg.2016.118 PMC5206767

[CR45] Hsieh J, Koseki M, Molusky MM, Yakushiji E, Ichi I, Westerterp M, et al. TTC39B deficiency stabilizes LXR reducing both atherosclerosis and steatohepatitis. Nature. 2016;535(7611):303–7. 10.1038/nature18628. 27383786 10.1038/nature18628 PMC4947007

[CR46] Jackson AL, Bartz SR, Schelter J, Kobayashi SV, Burchard J, Mao M, et al. Expression profiling reveals off-target gene regulation by RNAi. Nat Biotechnol. 2003;21(6):635–7. 10.1038/nbt831. 12754523 10.1038/nbt831

[CR47] Jin X, Xie H, Liu X, Shen Q, Wang Z, Hao H, et al. RELL1, a novel oncogene, accelerates tumor progression and regulates immune infiltrates in glioma. Int Immunopharmacol. 2020;87:106707. 10.1016/j.intimp.2020.106707. 32683297 10.1016/j.intimp.2020.106707

[CR48] Jung M, Peterson H, Chavez L, Kahlem P, Lehrach H, Vilo J, et al. A data integration approach to mapping OCT4 gene regulatory networks operative in embryonic stem cells and embryonal carcinoma cells. PLoS ONE. 2010;5(5):e10709. 10.1371/journal.pone.0010709. 20505756 10.1371/journal.pone.0010709 PMC2873957

[CR49] Kaelin WG. Use and abuse of RNAi to study mammalian gene function. Science. 2012;337(6093):421–2. 10.1126/science.1225787. 22837515 10.1126/science.1225787 PMC3705935

[CR50] Khan FA, Fang N, Zhang W, Ji S. The multifaceted role of Fragile X-Related Protein 1 (FXR1) in cellular processes: an updated review on cancer and clinical applications. Cell Death Dis. 2024;15(1):72. 10.1038/s41419-023-06413-8. 38238286 10.1038/s41419-023-06413-8 PMC10796922

[CR51] Kim J, Chu J, Shen X, Wang J, Orkin SH. An extended transcriptional network for pluripotency of embryonic stem cells. Cell. 2008;132(6):1049–61. 10.1016/j.cell.2008.02.039. 18358816 10.1016/j.cell.2008.02.039 PMC3837340

[CR52] Kotkamp K, Mössner R, Allen A, Onichtchouk D, Driever W. A *Pou5f1*/Oct4 dependent *Klf2a*, *Klf2b*, and *Klf17* regulatory sub-network contributes to EVL and ectoderm development during zebrafish embryogenesis. Dev Biol. 2014;385(2):433–47. 10.1016/j.ydbio.2013.10.025. 24211655 10.1016/j.ydbio.2013.10.025

[CR53] Kuroda T, Tada M, Kubota H, Kimura H, Hatano SY, Suemori H, et al. Octamer and Sox elements are required for transcriptional cis regulation of Nanog gene expression. Mol Cell Biol. 2005;25(6):2475–85. 10.1128/mcb.25.6.2475-2485.2005. 15743839 10.1128/MCB.25.6.2475-2485.2005 PMC1061601

[CR54] Lennartsson J, Rönnstrand L. Stem cell factor receptor/c-Kit: from basic science to clinical implications. Physiol Rev. 2012;92(4):1619–49. 10.1152/physrev.00046.2011. 23073628 10.1152/physrev.00046.2011

[CR55] Leong WZ, Tan SH, Ngoc PCT, Amanda S, Yam AWY, Liau WS, et al. ARID5B as a critical downstream target of the TAL1 complex that activates the oncogenic transcriptional program and promotes T-cell leukemogenesis. Genes Dev. 2017;31(23–24):2343–60. 10.1101/gad.302646.117. 29326336 10.1101/gad.302646.117 PMC5795782

[CR56] Li P, Ma X, Adams IR, Yuan P. A tight control of Rif1 by Oct4 and Smad3 is critical for mouse embryonic stem cell stability. Cell Death Dis. 2015;6(1):e1588. 10.1038/cddis.2014.551. 25569105 10.1038/cddis.2014.551 PMC4669749

[CR57] Li W, Notani D, Rosenfeld MG. Enhancers as non-coding RNA transcription units: recent insights and future perspectives. Nat Rev Genet. 2016;17(4):207–23. 10.1038/nrg.2016.4. 26948815 10.1038/nrg.2016.4

[CR58] Li J, Dai C, Xie W, Zhang H, Huang X, Chronis C, et al. A one-step strategy to target essential factors with auxin-inducible degron system in mouse embryonic stem cells. Front Cell Dev Biol. 2022;10:964119. 10.3389/fcell.2022.964119. 36003152 10.3389/fcell.2022.964119 PMC9393215

[CR59] Liu X, Huang J, Chen T, Wang Y, Xin S, Li J, et al. Yamanaka factors critically regulate the developmental signaling network in mouse embryonic stem cells. Cell Res. 2008;18(12):1177–89. 10.1038/cr.2008.309. 19030024 10.1038/cr.2008.309

[CR60] Liu Y, Kim H, Liang J, Lu W, Ouyang B, Liu D, et al. The death-inducer obliterator 1 (Dido1) gene regulates embryonic stem cell self-renewal. J Biol Chem. 2014;289(8):4778–86. 10.1074/jbc.M113.486290. 24347171 10.1074/jbc.M113.486290 PMC3931039

[CR61] Liu Y, Lai S, Ma W, Ke W, Zhang C, Liu S, et al. CDYL suppresses epileptogenesis in mice through repression of axonal Nav1.6 sodium channel expression. Nat Commun. 2017;8(1):355. 10.1038/s41467-017-00368-z. 28842554 10.1038/s41467-017-00368-z PMC5572458

[CR62] Liu R, Jagannathan R, Sun L, Li F, Yang P, Lee J, et al. Tead1 is essential for mitochondrial function in cardiomyocytes. Am J Physiol Heart Circ Physiol. 2020;319(1):H89-h99. 10.1152/ajpheart.00732.2019. 32502376 10.1152/ajpheart.00732.2019 PMC7474438

[CR63] Liu LJ, Lv Z, Xue X, Xing ZY, Zhu F. Canonical WNT signaling activated by WNT7B contributes to L-HBs-mediated sorafenib resistance in hepatocellular carcinoma by inhibiting mitophagy. Cancers (Basel). 2022. 10.3390/cancers14235781. 36497264 10.3390/cancers14235781 PMC9741164

[CR64] Livak KJ, Schmittgen TD. Analysis of relative gene expression data using real-time quantitative PCR and the 2(-Delta Delta C(T)) Method. Methods (San Diego, Calif). 2001;25(4):402–8. 10.1006/meth.2001.1262. 11846609 10.1006/meth.2001.1262

[CR65] Livigni A, Peradziryi H, Sharov AA, Chia G, Hammachi F, Migueles RP, et al. A conserved Oct4/POUV-dependent network links adhesion and migration to progenitor maintenance. Curr Biol. 2013;23(22):2233–44. 10.1016/j.cub.2013.09.048. 24210613 10.1016/j.cub.2013.09.048 PMC4228055

[CR66] Loh YH, Wu Q, Chew JL, Vega VB, Zhang W, Chen X, et al. The Oct4 and Nanog transcription network regulates pluripotency in mouse embryonic stem cells. Nat Genet. 2006;38(4):431–40. 10.1038/ng1760. 16518401 10.1038/ng1760

[CR67] Loh YH, Zhang W, Chen X, George J, Ng HH. Jmjd1a and Jmjd2c histone H3 lys 9 demethylases regulate self-renewal in embryonic stem cells. Genes Dev. 2007;21(20):2545–57. 10.1101/gad.1588207. 17938240 10.1101/gad.1588207 PMC2000320

[CR68] Lv H, Yang J, Wang C, Yu F, Huang D, Ye L. The WNT7B protein promotes the migration and differentiation of human dental pulp cells partly through WNT/beta-catenin and c-Jun N-terminal kinase signalling pathways. Arch Oral Biol. 2018;87:54–61. 10.1016/j.archoralbio.2017.12.015. 29268145 10.1016/j.archoralbio.2017.12.015

[CR69] Ma H, Qu J, Pang Z, Luo J, Yan M, Xu W, et al. Super-enhancer omics in stem cell. Mol Cancer. 2024;23(1):153. 10.1186/s12943-024-02066-z. 39090713 10.1186/s12943-024-02066-z PMC11293198

[CR70] Markus-Koch A, Schmitt O, Seemann S, Lukas J, Koczan D, Ernst M, et al. ADAM23 promotes neuronal differentiation of human neural progenitor cells. Cell Mol Biol Lett. 2017;22:16. 10.1186/s11658-017-0045-1. 28828010 10.1186/s11658-017-0045-1 PMC5562998

[CR71] Meng S, Liu X, Zhu S, Xie P, Fang H, Pan Q, et al. Young LINE-1 transposon 5’ UTRs marked by elongation factor ELL3 function as enhancers to regulate naïve pluripotency in embryonic stem cells. Nat Cell Biol. 2023;25(9):1319–31. 10.1038/s41556-023-01211-y. 37591949 10.1038/s41556-023-01211-y

[CR72] Miki TS, Großhans H. The multifunctional RNase XRN2. Biochem Soc Trans. 2013;41(4):825–30. 10.1042/bst20130001. 23863139 10.1042/BST20130001

[CR73] Mora A, Sandve GK, Gabrielsen OS, Eskeland R. In the loop: promoter-enhancer interactions and bioinformatics. Brief Bioinform. 2016;17(6):980–95. 10.1093/bib/bbv097. 26586731 10.1093/bib/bbv097 PMC5142009

[CR74] Morawska M, Ulrich HD. An expanded tool kit for the auxin-inducible degron system in budding yeast. Yeast. 2013;30(9):341–51. 10.1002/yea.2967. 23836714 10.1002/yea.2967 PMC4171812

[CR75] Muhar M, Ebert A, Neumann T, Umkehrer C, Jude J, Wieshofer C, et al. SLAM-seq defines direct gene-regulatory functions of the BRD4-MYC axis. Science. 2018;360(6390):800–5. 10.1126/science.aao2793. 29622725 10.1126/science.aao2793 PMC6409205

[CR76] Närvä E, Rahkonen N, Emani MR, Lund R, Pursiheimo JP, Nästi J, et al. RNA-binding protein L1TD1 interacts with LIN28 via RNA and is required for human embryonic stem cell self-renewal and cancer cell proliferation. Stem Cells. 2012;30(3):452–60. 10.1002/stem.1013. 22162396 10.1002/stem.1013 PMC3507993

[CR77] Natsume T, Kiyomitsu T, Saga Y, Kanemaki MT. Rapid protein depletion in human cells by auxin-inducible degron tagging with short homology donors. Cell Rep. 2016;15(1):210–8. 10.1016/j.celrep.2016.03.001. 27052166 10.1016/j.celrep.2016.03.001

[CR78] Ng LY, Ma HT, Liu JCY, Huang X, Lee N, Poon RYC. Conditional gene inactivation by combining tetracycline-mediated transcriptional repression and auxin-inducible degron-mediated degradation. Cell Cycle. 2019;18(2):238–48. 10.1080/15384101.2018.1563395. 30582405 10.1080/15384101.2018.1563395 PMC6343694

[CR79] Ngubo M, Moradi F, Ito CY, Stanford WL. Tissue-specific tumour suppressor and oncogenic activities of the polycomb-like protein MTF2. Genes. 2023. 10.3390/genes14101879. 37895228 10.3390/genes14101879 PMC10606531

[CR80] Nishimura K, Fukagawa T, Takisawa H, Kakimoto T, Kanemaki M. An auxin-based degron system for the rapid depletion of proteins in nonplant cells. Nat Methods. 2009;6(12):917–22. 10.1038/nmeth.1401. 19915560 10.1038/nmeth.1401

[CR81] Nishimura K, Yamada R, Hagihara S, Iwasaki R, Uchida N, Kamura T, et al. A super-sensitive auxin-inducible degron system with an engineered auxin-TIR1 pair. Nucleic Acids Res. 2020;48(18):e108. 10.1093/nar/gkaa748. 32941625 10.1093/nar/gkaa748 PMC7544234

[CR82] Niwa H. Molecular mechanism to maintain stem cell renewal of ES cells. Cell Struct Funct. 2001;26(3):137–48. 10.1247/csf.26.137. 11565806 10.1247/csf.26.137

[CR83] Niwa H, Miyazaki J, Smith AG. Quantitative expression of Oct-3/4 defines differentiation, dedifferentiation or self-renewal of ES cells. Nat Genet. 2000;24(4):372–6. 10.1038/74199. 10742100 10.1038/74199

[CR84] Niwa H, Masui S, Chambers I, Smith AG, Miyazaki J. Phenotypic complementation establishes requirements for specific POU domain and generic transactivation function of Oct-3/4 in embryonic stem cells. Mol Cell Biol. 2002;22(5):1526–36. 10.1128/mcb.22.5.1526-1536.2002. 11839818 10.1128/mcb.22.5.1526-1536.2002 PMC134688

[CR85] Niwa H, Toyooka Y, Shimosato D, Strumpf D, Takahashi K, Yagi R, et al. Interaction between Oct3/4 and Cdx2 determines trophectoderm differentiation. Cell. 2005;123(5):917–29. 10.1016/j.cell.2005.08.040. 16325584 10.1016/j.cell.2005.08.040

[CR86] Nora EP, Goloborodko A, Valton AL, Gibcus JH, Uebersohn A, Abdennur N, et al. Targeted degradation of CTCF decouples local insulation of chromosome domains from genomic compartmentalization. Cell. 2017;169(5):930-44.e22. 10.1016/j.cell.2017.05.004. 28525758 10.1016/j.cell.2017.05.004 PMC5538188

[CR87] Ohbayashi N, Maruta Y, Ishida M, Fukuda M. Melanoregulin regulates retrograde melanosome transport through interaction with the RILP-p150Glued complex in melanocytes. J Cell Sci. 2012;125(Pt 6):1508–18. 10.1242/jcs.094185. 22275436 10.1242/jcs.094185

[CR88] Palozola KC, Donahue G, Zaret KS. EU-RNA-seq for in vivo labeling and high throughput sequencing of nascent transcripts. STAR Protoc. 2021;2(3):100651. 10.1016/j.xpro.2021.100651. 34485932 10.1016/j.xpro.2021.100651 PMC8403648

[CR89] Prieto-Garcia C, Tomašković I, Shah VJ, Dikic I, Diefenbacher M. USP28: oncogene or tumor suppressor? A unifying paradigm for squamous cell carcinoma. Cells. 2021. 10.3390/cells10102652. 34685632 10.3390/cells10102652 PMC8534253

[CR90] Prozzillo Y, Fattorini G, Santopietro MV, Suglia L, Ruggiero A, Ferreri D, et al. Targeted protein degradation tools: overview and future perspectives. Biology. 2020. 10.3390/biology9120421. 33256092 10.3390/biology9120421 PMC7761331

[CR91] Rodda DJ, Chew JL, Lim LH, Loh YH, Wang B, Ng HH, et al. Transcriptional regulation of nanog by OCT4 and SOX2. J Biol Chem. 2005;280(26):24731–7. 10.1074/jbc.M502573200. 15860457 10.1074/jbc.M502573200

[CR92] Rykaczewska U, Suur BE, Röhl S, Razuvaev A, Lengquist M, Sabater-Lleal M, et al. PCSK6 is a key protease in the control of smooth muscle cell function in vascular remodeling. Circ Res. 2020;126(5):571–85. 10.1161/circresaha.119.316063. 31893970 10.1161/CIRCRESAHA.119.316063

[CR93] Sacher R, Stergiou L, Pelkmans L. Lessons from genetics: interpreting complex phenotypes in RNAi screens. Curr Opin Cell Biol. 2008;20(4):483–9. 10.1016/j.ceb.2008.06.002. 18602470 10.1016/j.ceb.2008.06.002

[CR94] Sauer B, Henderson N. The cyclization of linear DNA in *Escherichia coli* by site-specific recombination. Gene. 1988;70(2):331–41. 10.1016/0378-1119(88)90205-3. 3063605 10.1016/0378-1119(88)90205-3

[CR95] Sevilla A, Grichnik J. Therapeutic modulation of KIT ligand in melanocytic disorders with implications for mast cell diseases. Exp Dermatol. 2024;33(5):e15091. 10.1111/exd.15091. 38711220 10.1111/exd.15091

[CR96] Sharov AA, Masui S, Sharova LV, Piao Y, Aiba K, Matoba R, et al. Identification of Pou5f1, Sox2, and Nanog downstream target genes with statistical confidence by applying a novel algorithm to time course microarray and genome-wide chromatin immunoprecipitation data. BMC Genomics. 2008;9:269. 10.1186/1471-2164-9-269. 18522731 10.1186/1471-2164-9-269 PMC2424064

[CR97] Shi M, Tie HC, Divyanshu M, Sun X, Zhou Y, Boh BK, et al. Arl15 upregulates the TGFβ family signaling by promoting the assembly of the Smad-complex. Elife. 2022. 10.7554/eLife.76146. 35834310 10.7554/eLife.76146 PMC9352346

[CR98] Shin J, Tkachenko S, Chaklader M, Pletz C, Singh K, Bulut GB, et al. Endothelial OCT4 is atheroprotective by preventing metabolic and phenotypic dysfunction. Cardiovasc Res. 2022;118(11):2458–77. 10.1093/cvr/cvac036. 35325071 10.1093/cvr/cvac036 PMC9890633

[CR99] Siouda M, Dujardin AD, Barbollat-Boutrand L, Mendoza-Parra MA, Gibert B, Ouzounova M, et al. CDYL2 epigenetically regulates MIR124 to control NF-κB/STAT3-dependent breast cancer cell plasticity. iScience. 2020;23(6):101141. 10.1016/j.isci.2020.101141. 32450513 10.1016/j.isci.2020.101141 PMC7251929

[CR100] Skalska L, Beltran-Nebot M, Ule J, Jenner RG. Regulatory feedback from nascent RNA to chromatin and transcription. Nat Rev Mol Cell Biol. 2017;18(5):331–7. 10.1038/nrm.2017.12. 28270684 10.1038/nrm.2017.12

[CR101] Sulistomo HW, Nemoto T, Yanagita T, Takeya R. Formin homology 2 domain-containing 3 (Fhod3) controls neural plate morphogenesis in mouse cranial neurulation by regulating multidirectional apical constriction. J Biol Chem. 2019;294(8):2924–34. 10.1074/jbc.RA118.005471. 30573686 10.1074/jbc.RA118.005471 PMC6393623

[CR102] Tan X, Calderon-Villalobos LI, Sharon M, Zheng C, Robinson CV, Estelle M, et al. Mechanism of auxin perception by the TIR1 ubiquitin ligase. Nature. 2007;446(7136):640–5. 10.1038/nature05731. 17410169 10.1038/nature05731

[CR103] Thomas MK, Tsang SW, Yeung ML, Leung PS, Yao KM. The roles of the PDZ-containing proteins bridge-1 and PDZD2 in the regulation of insulin production and pancreatic beta-cell mass. Curr Protein Pept Sci. 2009;10(1):30–6. 10.2174/138920309787315248. 19275670 10.2174/138920309787315248

[CR104] Tokuzawa Y, Kaiho E, Maruyama M, Takahashi K, Mitsui K, Maeda M, et al. Fbx15 is a novel target of Oct3/4 but is dispensable for embryonic stem cell self-renewal and mouse development. Mol Cell Biol. 2003;23(8):2699–708. 10.1128/mcb.23.8.2699-2708.2003. 12665572 10.1128/MCB.23.8.2699-2708.2003 PMC152544

[CR105] Toledo F, Wahl GM. MDM2 and MDM4: p53 regulators as targets in anticancer therapy. Int J Biochem Cell Biol. 2007;39(7–8):1476–82. 10.1016/j.biocel.2007.03.022. 17499002 10.1016/j.biocel.2007.03.022 PMC2043116

[CR106] Trost M, Blattner AC, Lehner CF. Regulated protein depletion by the auxin-inducible degradation system in *Drosophila melanogaster*. Fly. 2016;10(1):35–46. 10.1080/19336934.2016.1168552. 27010248 10.1080/19336934.2016.1168552 PMC4934730

[CR107] Wang X, Liu Z, Zhang L, Yang Z, Chen X, Luo J, et al. Targeting deubiquitinase USP28 for cancer therapy. Cell Death Dis. 2018;9(2):186. 10.1038/s41419-017-0208-z. 29415985 10.1038/s41419-017-0208-z PMC5833459

[CR108] Wang Y, Wang M, Zheng T, Hou Y, Zhang P, Tang T, et al. Specificity profiling of CRISPR system reveals greatly enhanced off-target gene editing. Sci Rep. 2020;10(1):2269. 10.1038/s41598-020-58627-x. 32042045 10.1038/s41598-020-58627-x PMC7010781

[CR109] Wang J, Miao Y, Wicklein R, Sun Z, Wang J, Jude KM, et al. RTN4/NoGo-receptor binding to BAI adhesion-GPCRs regulates neuronal development. Cell. 2021a;184(24):5869-85.e25. 10.1016/j.cell.2021.10.016. 34758294 10.1016/j.cell.2021.10.016 PMC8620742

[CR110] Wang J, Yu H, Ma Q, Zeng P, Wu D, Hou Y, et al. Phase separation of OCT4 controls TAD reorganization to promote cell fate transitions. Cell Stem Cell. 2021b;28(10):1868-83.e11. 10.1016/j.stem.2021.04.023. 34038708 10.1016/j.stem.2021.04.023

[CR111] Weiss WA, Taylor SS, Shokat KM. Recognizing and exploiting differences between RNAi and small-molecule inhibitors. Nat Chem Biol. 2007;3(12):739–44. 10.1038/nchembio1207-739. 18007642 10.1038/nchembio1207-739 PMC2924165

[CR112] Whyte WA, Orlando DA, Hnisz D, Abraham BJ, Lin CY, Kagey MH, et al. Master transcription factors and mediator establish super-enhancers at key cell identity genes. Cell. 2013;153(2):307–19. 10.1016/j.cell.2013.03.035. 23582322 10.1016/j.cell.2013.03.035 PMC3653129

[CR113] Wissink EM, Vihervaara A, Tippens ND, Lis JT. Nascent RNA analyses: tracking transcription and its regulation. Nat Rev Genet. 2019;20(12):705–23. 10.1038/s41576-019-0159-6. 31399713 10.1038/s41576-019-0159-6 PMC6858503

[CR114] Wood L, Booth DG, Vargiu G, Ohta S, deLima Alves F, Samejima K, et al. Auxin/AID versus conventional knockouts: distinguishing the roles of CENP-T/W in mitotic kinetochore assembly and stability. Open Biol. 2016;6(1):150230. 10.1098/rsob.150230. 26791246 10.1098/rsob.150230 PMC4736828

[CR115] Wu Y, Guo Z, Liu Y, Tang B, Wang Y, Yang L, et al. Oct4 and the small molecule inhibitor, SC1, regulates Tet2 expression in mouse embryonic stem cells. Mol Biol Rep. 2013;40(4):2897–906. 10.1007/s11033-012-2305-5. 23254757 10.1007/s11033-012-2305-5

[CR116] Xie F, Ye L, Ta M, Zhang L, Jiang WG. MTSS1: a multifunctional protein and its role in cancer invasion and metastasis. Front Biosci (Schol Ed). 2011;3(2):621–31. 10.2741/s175. 21196400 10.2741/s175

[CR117] Xiong B, Yang Y, Fineis FR, Wang JP. Degnorm: normalization of generalized transcript degradation improves accuracy in RNA-seq analysis. Genome Biol. 2019;20(1):75. 10.1186/s13059-019-1682-7. 30992037 10.1186/s13059-019-1682-7 PMC6466807

[CR118] Xiong L, Tolen EA, Choi J, Velychko S, Caizzi L, Velychko T, et al. Oct4 differentially regulates chromatin opening and enhancer transcription in pluripotent stem cells. Elife. 2022. 10.7554/eLife.71533. 35621159 10.7554/eLife.71533 PMC9142147

[CR119] Xu Z, Asakawa S. Physiological RNA dynamics in RNA-Seq analysis. Brief Bioinform. 2019;20(5):1725–33. 10.1093/bib/bby045. 30010714 10.1093/bib/bby045

[CR120] Yan Y, Tan MW, Xue X, Ding XY, Wang GK, Xu ZY. Involvement of Oct4 in the pathogenesis of thoracic aortic dissection via inducing the dedifferentiated phenotype of human aortic smooth muscle cells by directly upregulating KLF5. J Thorac Cardiovasc Surg. 2016;152(3):820-9.e4. 10.1016/j.jtcvs.2016.05.036. 27353340 10.1016/j.jtcvs.2016.05.036

[CR121] Yang HW, Kim TM, Song SS, Menon L, Jiang X, Huang W, et al. A small subunit processome protein promotes cancer by altering translation. Oncogene. 2015;34(34):4471–81. 10.1038/onc.2014.376. 25435373 10.1038/onc.2014.376

[CR122] Yang K, Luan YY, Wang S, Yan YS, Wang YP, Wu J, et al. SGMS1 facilitates osteogenic differentiation of MSCs and strengthens osteogenesis-angiogenesis coupling by modulating Cer/PP2A/Akt pathway. iScience. 2024;27(4):109358. 10.1016/j.isci.2024.109358. 38544565 10.1016/j.isci.2024.109358 PMC10966191

[CR123] Yao Y, Zhou S, Yan Y, Fu K, Xiao S. The tripartite motif-containing 24 is a multifunctional player in human cancer. Cell Biosci. 2024;14(1):103. 10.1186/s13578-024-01289-3. 39160596 10.1186/s13578-024-01289-3 PMC11334367

[CR124] Ye Y, Chen X, Zhang W. Mammalian SWI/SNF chromatin remodeling complexes in embryonic stem cells: regulating the balance between pluripotency and differentiation. Front Cell Dev Biol. 2020;8:626383. 10.3389/fcell.2020.626383. 33537314 10.3389/fcell.2020.626383 PMC7848206

[CR125] Yi X, Li M, He G, Du H, Li X, Cao D, et al. Genetic and functional analysis reveals TENM4 contributes to schizophrenia. iScience. 2021;24(9):103063. 10.1016/j.isci.2021.103063. 34568788 10.1016/j.isci.2021.103063 PMC8449235

[CR126] You KT, Park J, Kim VN. Role of the small subunit processome in the maintenance of pluripotent stem cells. Genes Dev. 2015;29(19):2004–9. 10.1101/gad.267112.115. 26443847 10.1101/gad.267112.115 PMC4604342

[CR127] Young RA. Control of the embryonic stem cell state. Cell. 2011;144(6):940–54. 10.1016/j.cell.2011.01.032. 21414485 10.1016/j.cell.2011.01.032 PMC3099475

[CR128] Yu ZH, Wang YM, Jiang YZ, Ma SJ, Zhong Q, Wan YY, et al. NID2 can serve as a potential prognosis prediction biomarker and promotes the invasion and migration of gastric cancer. Pathology. 2019;215(10):152553. 10.1016/j.prp.2019.152553. 10.1016/j.prp.2019.152553 31362888

[CR129] Zafarana G, Avery SR, Avery K, Moore HD, Andrews PW. Specific knockdown of OCT4 in human embryonic stem cells by inducible short hairpin RNA interference. Stem Cells. 2009;27(4):776–82. 10.1002/stem.5. 19350677 10.1002/stem.5 PMC2847189

[CR130] Zhang L, Ward JD, Cheng Z, Dernburg AF. The auxin-inducible degradation (AID) system enables versatile conditional protein depletion in C. elegans. Development. 2015;142(24):4374–84. 10.1242/dev.129635. 26552885 10.1242/dev.129635 PMC4689222

[CR131] Zhang W, Chronis C, Chen X, Zhang H, Spalinskas R, Pardo M, et al. The BAF and PRC2 Complex Subunits Dpf2 and Eed Antagonistically Converge on Tbx3 to Control ESC Differentiation. Cell Stem Cell. 2019;24(1):138-52.e8. 10.1016/j.stem.2018.12.001. 30609396 10.1016/j.stem.2018.12.001 PMC6486830

[CR132] Zhang J, Zhou Y, Yue W, Zhu Z, Wu X, Yu S, et al. Super-enhancers conserved within placental mammals maintain stem cell pluripotency. Proc Natl Acad Sci U S A. 2022a;119(40):e2204716119. 10.1073/pnas.2204716119. 36161929 10.1073/pnas.2204716119 PMC9546576

[CR133] Zhang X, Tian L, Li Z, Liu R, Yu J, Liu B. CAMK2N1 has a cancer-suppressive function in colorectal carcinoma via effects on the Wnt/β-catenin pathway. Biochem Biophys Res Commun. 2022b;626:220–8. 10.1016/j.bbrc.2022.08.036. 35998547 10.1016/j.bbrc.2022.08.036

[CR134] Zhao L, Liu S, Peng Y, Zhang J. Lamc1 promotes osteogenic differentiation and inhibits adipogenic differentiation of bone marrow-derived mesenchymal stem cells. Sci Rep. 2024;14(1):19592. 10.1038/s41598-024-69629-4. 39179716 10.1038/s41598-024-69629-4 PMC11344058

[CR135] Zhou RM, Shi LJ, Shan K, Sun YN, Wang SS, Zhang SJ, et al. Circular RNA-ZBTB44 regulates the development of choroidal neovascularization. Theranostics. 2020a;10(7):3293–307. 10.7150/thno.39488. 32194869 10.7150/thno.39488 PMC7053208

[CR136] Zhou Y, Cao G, Cai H, Huang H, Zhu X. The effect and clinical significance of FN1 expression on biological functions of gastric cancer cells. Cell Mol Biol. 2020;66(5):191–8. 10.14715/cmb/2020.66.5.32. 33040835

