# Supplementary figures and images for: Systematic identification of Oct4 transcriptional targets in embryonic stem cells using the auxin-inducible degron system and nascent RNA sequencing
Source: Cell Regen. 2025 Dec 3;14:49. doi: 10.1186/s13619-025-00269-3 (PMC12675901; doi:10.1186/s13619-025-00269-3)

**A****4h down**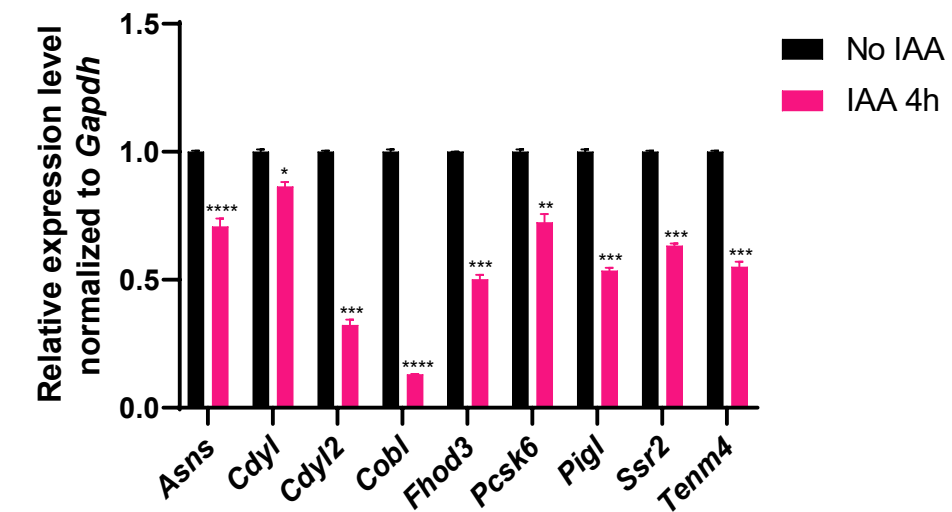**C****4h up**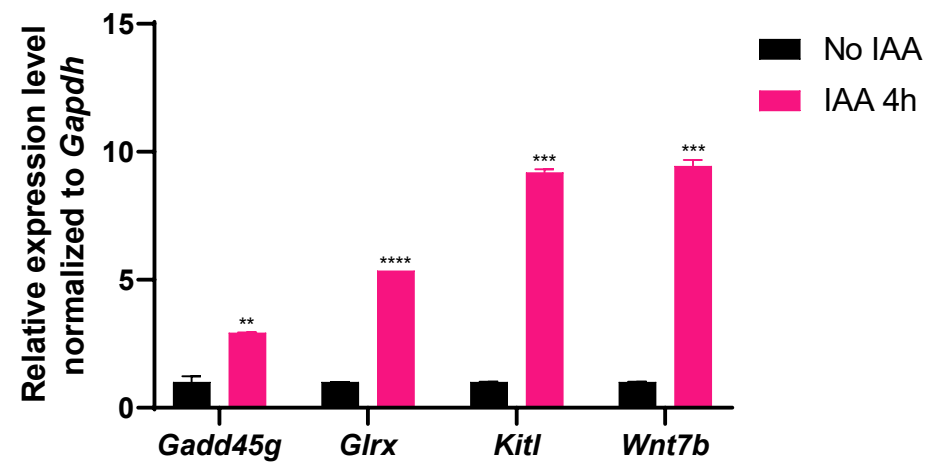**B****24h down**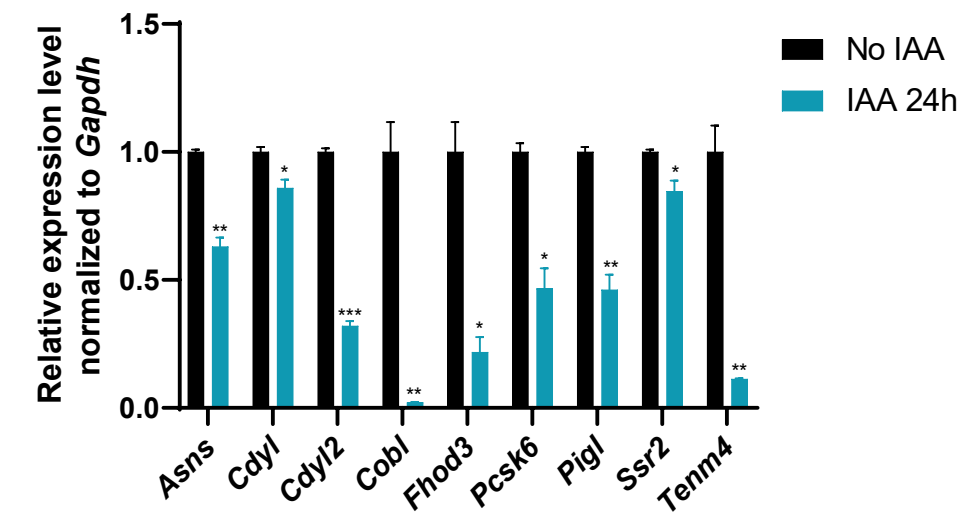**D****24h up**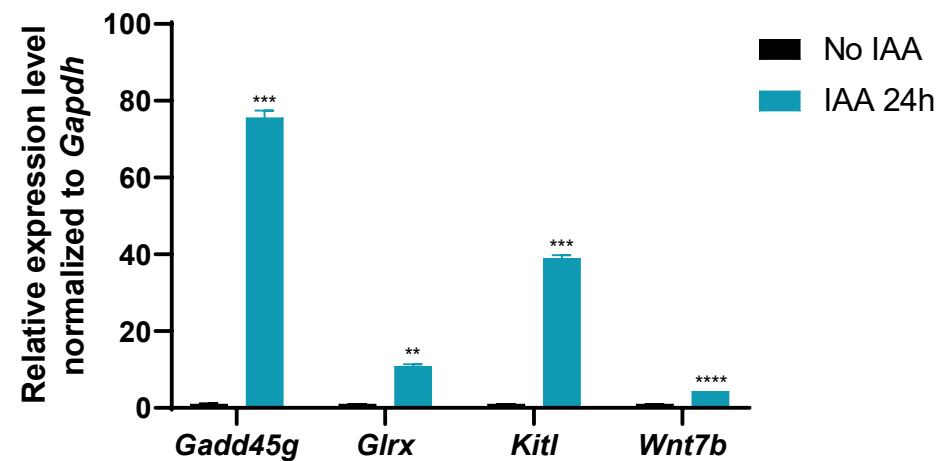

Supplement: Supplementary file 1 — Supplementary Material 1. Fig. S1 qPCR validation of putative Oct4 target genes. A-B Relative transcript levels of the indicated downregulated Oct4 target genes were measured by qPCR in Oct4-mAID ESCs after 4 h (A) and 24 h (B) of IAA treatment. C-D Transcriptional levels of the indicated upregulated target genes were measured by qPCR in Oct4-mAID ESCs after 4 h (C) and 24 h (D) of IAA treatment. Gene expression was normalized to Gapdh and presented relative to untreated controls. Statistical significance was assessed by two-tailed unpaired t-test; *P < 0.05, **P < 0.01, ***P < 0.001, ****P < 0.0001. [file 13619_2025_269_MOESM1_ESM.pdf]
