# Supplementary material for: Systematic identification of Oct4 transcriptional targets in embryonic stem cells using the auxin-inducible degron system and nascent RNA sequencing
Source: Cell Regen. 2025 Dec 3;14:49. doi: 10.1186/s13619-025-00269-3 (PMC12675901; doi:10.1186/s13619-025-00269-3)
Supplement: Supplementary file 5 — Supplementary Material 5. Table S3A. Summary of Oct4 putative direct targets reported in the literature. Table S3B. Newly identified potential direct targets of Oct4 [Akerberg et al. 2022; Antao et al. 2021; Aygün et al. 2021; Berger et al. 2024; Bygrave et al. 2023; Cai et al. 2022; Chen et al. 2022a; Cooke et al. 2019; Deng et al. 2022; Desfougères et al. 2019; Gatchalian et al. 2018; He et al. 2023; Hsieh et al. 2016; Jin et al. 2020; Khan et al. 2024; Lennartsson et al. 2012; Leong et al. 2017; Liu et al. 2017; Liu et al. 2020; Markus-Koch et al. 2017; Miki and Großhans 2013; Ngubo et al. 2023; Ohbayashi et al. 2012; Prieto-Garcia et al. 2021; Rykaczewska et al. 2020; Sevilla and Grichnik 2024; Shi et al. 2022; Siouda et al. 2020; Sulistomo et al. 2019; Thomas et al.2009; Toledo et al. 2007; Wang et al. 2018; Wang et al. 2021; Xie et al. 2011; Yang et al. 2015; Yang et al. 2024; Yao et al. 2024; Yi et al. 2021; Yu et al. 2019; Zhao et al. 2024; Zhou et al. 2020; Zhou et al. 2020]. [file 13619_2025_269_MOESM5_ESM.docx]

Summary of Oct4 putative direct targets reported in the literature

| **Genes** | **log2FoldChange** | | **References** |
| --- | --- | --- | --- |
|  | **4 h** **v****s 0 h** | **24 h vs 0 h** | **(Oct4-regulated)** |
| *Nanog* | -1.50 | -2.49 | Oct4 and Sox2 were shown to regulate *Nanog* expression by synergistic binding to *Nanog* Oct-Sox motif.(Kuroda et al., 2005; Rodda et al., 2005) |
| *Esrrb* | -1.13 | -4.56 | *Rcor2*, *Esrrb* and *Phc1* are examples of transcriptional regulators positively regulated by both Oct4 and Nanog.(Loh et al., 2006) *Esrrb*, *Rif1* and *REST* are genes shown to be regulated by both Oct4 and Nanog.(Loh et al., 2006) |
| *Klf2* | -2.39 | -1.52 | In mammalian ES cells, the transcription factors Klf2 and Klf4 contribute to maintenance of pluripotency and self-renewal and are regulated by Pou5f1/Oct4.(Kotkamp et al., 2014) |
| *Klf4* | -3.17 | -4.12 |  |
| *Klf5* | -1.63 | -2.81 | Kruppel-like factor 5 (KLF5) was found to be a directly regulated target gene of Oct4 in HASMCs.(Yan et al., 2016) |
| *Dido1* | -2.42 | -2.84 | Dido1 is the target of canonical transcription factors such as Oct4, Sox2, and Nanog.(Liu et al., 2014) |
| *L1td1* | -2.49 | -3.91 | OCT4, NANOG and SOX2 could regulate expression of L1TD1.(Närvä et al., 2012) OCT4, SOX2 and NANOG all bind to the L1TD1 promoter in hESCs.(Närvä et al., 2012) |
| *Phc1* | -2.52 | -3.04 | *Phc1* and *Nanog* are direct transcriptional targets of Oct4.(Chen et al., 2021) *Phc1*, similar to *Nanog*, is transcriptionally activated by Oct4.(Chen et al., 2021) |
| *Zfp42 (*Rex1*)* | -2.72 | -4.43 | Rex-1 is regulated via Oct-3/4 and Oct-6 binding to an octamer site.(Ben-Shushan et al., 1998; Hosler et al., 1993) |
| *Tet2* | -2.74 | -4.20 | Oct4 directly regulates the expression of Tet2.(Wu et al., 2013) |
| *Kat6b* | -1.77 | -2.90 | Kat6b is regulated by the pluripotency transcription factors Nanog and Oct4.(Cosentino et al., 2019) |
| *Kdm3a (Jmjd1a)* | -1.16 | -1.47 | Oct4 binds to the intronic regions of *Jmjd1a* and *Jmjd2c.*(Loh et al., 2007) In ES cells, Oct4 up-regulates the levels of Jmjd1a and Jmjd2c.(Loh et al., 2007) |
| *Kdm4c (Jmjd2c)* | -1.20 | -2.03 |  |
| *Fbxo15 (Fbx15)* | -1.65 | -2.72 | Fbx15 is a novel target of Oct3/4 but is dispensable for embryonic stem cell self-renewal and mouse development.(Tokuzawa et al., 2003) |
| *Rif1* | -1.48 | -2.48 | Oct4 positively regulates *Rif1* expression in mouse ESCs.(Li et al., 2015) *Esrrb*, *Rif1* and *REST* are genes shown to be regulated by both Oct4 and Nanog.(Loh et al., 2006) |
| *Rest* | -1.10 | -1.61 | *Rest* is a direct Oct4 target.(Campbell et al., 2007) *Esrrb*, *Rif1* and *REST* are genes shown to be regulated by both Oct4 and Nanog.(Loh et al., 2006) |
| *Gadd45g* | 2.99 | 4.87 | *GADD45G* were found to be bound and regulated by OCT4.(Jung et al., 2010; Sharov et al., 2008) |
| …… | …… | …… | …… |

**Newly identified potential direct targets of Oct4**

| **Genes** | **log2FoldChange** | | **References** |
| --- | --- | --- | --- |
|  | **4 h vs 0 h** | **24 h vs 0 h** | **(Gene functions)** |
| *Cobl* | -3.70 | -5.51 | Involved in actin regulation and neuronal morphogenesis.(Ahuja et al., 2007) |
| *Pigl* | -2.15 | -3.32 | Involved in metabolism of proteins and post-translational modification: synthesis of GPI-anchored proteins.(Álvarez-Sánchez et al., 2024) |
| *Ssr2* | -2.05 | -3.32 | Involved in protein folding and processing.(Chen et al., 2022a) |
| *Pcsk6* | -1.72 | -3.09 | Involved in vascular remodeling.(Rykaczewska et al., 2020) |
| *Fbrsl1* | -1.69 | -2.12 | It is required for heart development.(Berger et al., 2024) |
| *Asns* | -1.67 | -3.42 | Regulates lung-cancer metastasis depending on Wnt pathway and mitochondrial functions.(Cai et al., 2022) |
| *Cdyl2* | -1.67 | -3.02 | It positively regulated breast cancer cell migration, invasion, stem-like phenotypes, and epithelial-to-mesenchymal transition.(Siouda et al., 2020) |
| *Fhod3* | -1.63 | -3.01 | It plays a crucial role in the morphological changes associated with neural tube closure at the hindbrain.(Sulistomo et al., 2019) |
| *Cdyl* | -1.57 | -2.59 | It is a critical player for experience-dependent gene regulation in controlling intrinsic excitability.(Liu et al., 2017) |
| *Tenm4* | -1.54 | -3.23 | It is highly expressed in the nervous system.(Yi et al., 2021) It is a risk gene shared by many types of mental diseases and is implicated in neuronal plasticity and signaling.(Yi et al., 2021) |
| *Mtss1* | -1.48 | -1.72 | It plays an inhibitory role in tumorigenesis and metastasis of a variety of cancers.(Chen et al., 2022b; Xie et al., 2011) |
| *Mtf2* | -1.32 | -2.40 | Regulates histone methylation and transcription.(Ngubo et al., 2023) |
| *Arl15* | -1.30 | -2.33 | It positively regulates the TGFβ family signaling.(Shi et al., 2022) |
| *Pdzd2* | -1.24 | -1.82 | It likely transduces signals that regulate insulin production, proliferation, and survival of pancreatic beta cells.(Thomas et al., 2009) |
| *Bicral* | -1.19 | -1.92 | BRD9 and GLTSCR1/BICRA or its paralog GLTSCR1-like/BICRAL define a non-canonical BAF complex that regulates naive pluripotency in mouse embryonic stem cells.(Gatchalian et al., 2018) |
| *Adam23* | -1.18 | -2.74 | Involved in neuronal differentiation.(Markus-Koch et al., 2017) |
| *Xrn2* | -1.18 | -1.75 | Involved in RNA metabolism regulation.(Aygün and Miki, 2021; Miki and Großhans, 2013) |
| *Usp48* | -1.17 | -1.72 | Involved in cell cycle regulation.(Antao et al., 2021) |
| *Ttc39b* | -1.16 | -2.31 | *Ttc39b* inhibition could be an effective strategy for reducing both steatohepatitis and atherosclerosis.(Hsieh et al., 2016) |
| *Rbpms2* | -1.16 | -1.95 | RBPMS2 is a myocardial-enriched splicing regulator required for cardiac function.(Akerberg et al., 2022) |
| *Mreg* | -1.15 | -2.64 | Involved in regulation of melanosome transport in mammalian epidermal melanocytes.(Ohbayashi et al., 2012) |
| *Enah* | -1.15 | -1.96 | Exacerbates the proliferation, invasion and migration of hepatocellular carcinoma cells via Notch signaling pathway.(Deng et al., 2022) |
| *Itpk1* | -1.12 | -1.75 | ITPK1 mediates the lipid-independent synthesis of inositol phosphates controlled by metabolism.(Desfougères et al., 2019) |
| *Fxr1* | -1.12 | -1.99 | Involved in RNA metabolism regulation.(Khan et al., 2024) |
| *Ttc7b* | -1.11 | -1.56 | It is closely associated with focal adhesions, immune infiltration, and ferroptosis.(He et al., 2023) |
| *Rell1* | -1.11 | -1.39 | A novel oncogene, accelerates tumor progression and regulates immune infiltrates in glioma.(Jin et al., 2020) |
| *Ybx3* | -1.11 | -1.76 | Emerges as a key regulator of amino acid levels.(Cooke et al., 2019) |
| *Tead1* | -1.11 | -1.11 | Involved in Hippo pathway and regulation of mitochondrial function.(Liu et al., 2020) |
| *Nid2* | -1.10 | -2.58 | Promotes the invasion and migration of gastric cancer.(Yu et al., 2019) |
| *Rtn4* | -1.09 | -1.31 | Regulates dendritic arborization, axonal elongation, and synapse formation.(Wang et al., 2021) |
| *Zbtb44* | -1.08 | -2.67 | Circular RNA-ZBTB44 regulates the development of choroidal neovascularization.(Zhou et al., 2020a) |
| *Trim24* | -1.07 | -1.60 | Regulation of cancer cell proliferation.(Yao et al., 2024) |
| *Usp28* | -1.06 | -2.01 | Regulate the ubiquitination status of several targets involved in proliferation, DNA repair, apoptosis and oncogenesis.(Prieto-Garcia et al., 2021; Wang et al., 2018) |
| *Fn1* | -1.06 | -2.13 | Regulation of cancer cell proliferation.(Zhou et al., 2020b) |
| *Sgms1* | -1.05 | -1.88 | Facilitates osteogenic differentiation of MSCs and strengthens osteogenesis-angiogenesis coupling.(Yang et al., 2024) |
| *Mdm4* | -1.05 | -1.82 | Regulates p53 activity.(Toledo and Wahl, 2007) |
| *Utp18* | -1.05 | -1.49 | It is frequently gained and overexpressed in cancer.(Yang et al., 2015) Alters translation to promote stress resistance and growth.(Yang et al., 2015) |
| *Lamc1* | -1.05 | -1.89 | Promotes osteogenic differentiation and inhibits adipogenic differentiation of bone marrow-derived mesenchymal stem cells.(Zhao et al., 2024) |
| *Arid5b* | -1.03 | -2.22 | Activates the oncogenic transcriptional program and promotes T-cell leukemogenesis.(Leong et al., 2017) |
| *Btbd11* | -1.01 | -1.90 | Supports cell-type-specific synaptic function.(Bygrave et al., 2023) |
| *Kitl* | 1.35 | 1.43 | Also known as stem cell factor (SCF).(Lennartsson and Rönnstrand, 2012) KIT ligand and its associated receptor KIT serve as a master regulatory system for both melanocytes and mast cells controlling survival, migration, proliferation and activation.(Sevilla and Grichnik, 2024) |
| *Glrx* | 1.24 | 0.20 | It is closely related to the tumor immune microenvironment.(Chang et al., 2020) |
| *Wnt7b* | 1.20 | -0.56 | Involved in regulating the Wnt signaling pathway.(Liu et al., 2022; Lv et al., 2018) |
| *Camk2n1* | 1.01 | -0.61 | It has a cancer-suppressive function.(Zhang et al., 2022) |
| …… | …… | …… | …… |
