# Supplementary material for: Systematic identification of Oct4 transcriptional targets in embryonic stem cells using the auxin-inducible degron system and nascent RNA sequencing
Source: Cell Regen. 2025 Dec 3;14:49. doi: 10.1186/s13619-025-00269-3 (PMC12675901; doi:10.1186/s13619-025-00269-3)
Supplement: Supplementary file 7 — Supplementary Material 7. Table S5A. Antibody information for Western blot. Table S5B. Primers for qPCR [file 13619_2025_269_MOESM7_ESM.docx]

**Antibody information for Western blot**

| **Antibodies** | **Source** | **Identifier** | **Dilution** |
| --- | --- | --- | --- |
| **OCT4** | SANTA CRUZ BIOTECHNOLOGY | sc-5279 | 1:3000 |
| **β-ACTIN** | SANTA CRUZ BIOTECHNOLOGY | sc-47778 | 1:1000 |

**Primers for qPCR**

| **Targeted Gene** | **Forward Primer (5´→ 3´)** | **Reverse Primer (5´→ 3´)** |
| --- | --- | --- |
| ***Gapdh*** | AGAACATCATCCCTGCATCC | CACATTGGGGGTAGGAACAC |
| ***Nanog*** | TCTTCCTGGTCCCCACAGTTT | GCAAGAATAGTTCTCGGGATGAA |
| ***Sox2*** | GCGGAGTGGAAACTTTTGTCC | CGGGAAGCGTGTACTTATCCTT |
| ***Klf4*** | GTGCCCCGACTAACCGTTG | GTCGTTGAACTCCTCGGTCT |
| ***Esrrb*** | GCACCTGGGCTCTAGTTGC | TACAGTCCTCGTAGCTCTTGC |
| ***Cdx2*** | CAAGGACGTGAGCATGTATCC | GTAACCACCGTAGTCCGGGTA |
| ***Fgf5*** | AAGTAGCGCGACGTTTTCTTC | CTGGAAACTGCTATGTTCCGAG |
| ***Asns*** | TTACCTGTCTCTGCCGCCAGAT | CACTGAAGGCTTCTTTGGGTCG |
| ***Cdyl*** | GCCTCACAGATGACCGAAAGAG | GGATGCTCCTAGTCCAATGGCT |
| ***Cdyl2*** | TGGCTGTTCCTCCTACACCTTC | CCAGAAGACCTGTGACACAAGG |
| ***Cobl*** | GGCAGAACTTGATGAAGACCTGG | GGCTTCTTGCAGACTGTGGTTG |
| ***Fhod3*** | GTTCTGCTTCCAGCATCTCGTC | CTGAAGGCTGGTGGACCACAAA |
| ***Pcsk6*** | CAGGCGCGAAGTGACTCTC | GACCGACAGCGACTGTTCTT |
| ***Pigl*** | GCAGAAGGAGTCAGTGGTCACA | ACTTCCGAAGCGCGTTCACAGA |
| ***Ssr2*** | CCTCCAGAAGACTTCGGCATTG | AGTAGCCAGCTTTGAGAGGACG |
| ***Tenm4*** | GGAATCATCTCCACTTTGCTGGG | GGTTGATGGCTAAGTCTGTGGG |
| ***Gadd45g*** | TCTACGAGTCCGCCAAAGTCCT | CTCACAGCAGAACGCCTGAATC |
| ***Glrx*** | GTTTCCTGAGTCATGCATCGGC | AGGTTGAGGCTGAGAACACTGG |
| ***Kitl*** | ATCTGCGGGAATCCTGTGACTG | CCATATCTCGTAGCCAACAATGAC |
| ***Wnt7b*** | TTCTCGTCGCTTTGTGGATGCC | CACCGTGACACTTACATTCCAGC |
