# Supplementary material for: Systematic identification of Oct4 transcriptional targets in embryonic stem cells using the auxin-inducible degron system and nascent RNA sequencing
Source: Cell Regen. 2025 Dec 3;14:49. doi: 10.1186/s13619-025-00269-3 (PMC12675901; doi:10.1186/s13619-025-00269-3)
Supplement: Supplementary file 2 — Supplementary Material 2. Fig. S2 Comparative transcriptomic profiling following OCT4 depletion in Oct4-mAID and Oct4f/- ESCs. A-B MA plots showing global gene expression changes in Oct4f/- ESCs following 4-OHT treatment for 12 h (A) and 24 h (B) (Bates et al., 2021). C-D MA plots depicting transcriptomic changes in Oct4-mAID ESCs upon IAA treatment for 4 h (C) and 24 h (D). Differentially expressed genes (fold change > 2, P-adj < 0.05) are highlighted in green. Representative pluripotency-associated genes are marked in blue, and differentiation-associated genes in red. E-F Venn diagrams showing the overlap of differentially expressed genes between Oct4f/- and Oct4-mAID ESCs. (E) Overlap of downregulated genes after 24 h 4-OHT treatment in Oct4f/- ESCs and those downregulated after 4 h IAA treatment in Oct4-mAID ESCs. (F) Overlap of downregulated genes following 24 h treatments in both systems. (Differential expression defined as fold change > 2, P-adj < 0.05). [file 13619_2025_269_MOESM2_ESM.pdf]

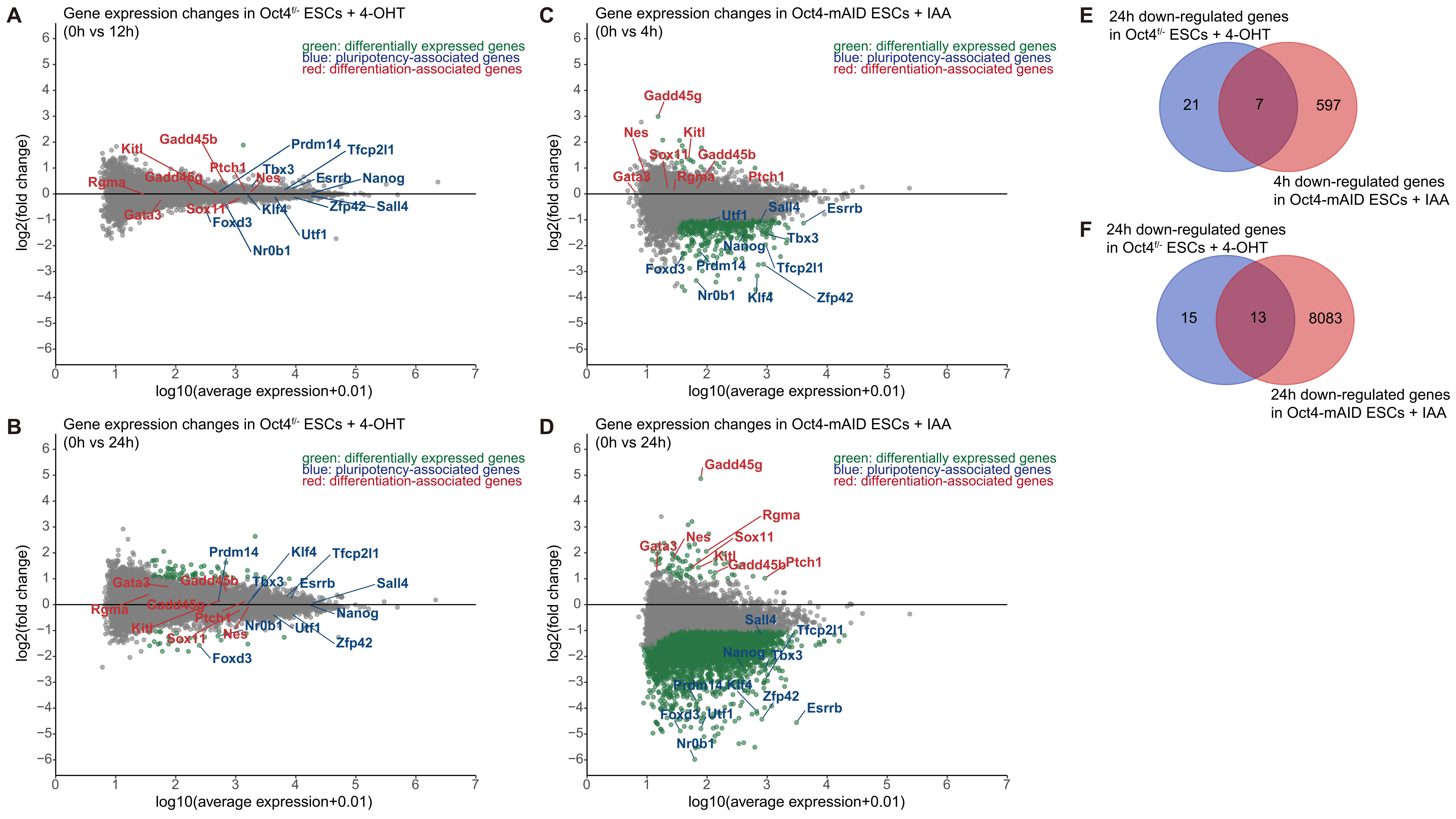
A
Gene expression changes in Oct4f/- ESCs + 4-OHT (0h vs 12h)
B
Gene expression changes in Oct4f/- ESCs + 4-OHT (0h vs 24h)
C
Gene expression changes in Oct4-mAID ESCs + IAA (0h vs 4h)
D
Gene expression changes in Oct4-mAID ESCs + IAA (0h vs 24h)
green: differentially expressed genes
blue: pluripotency-associated genes
red: differentiation-associated genes
log2(fold change)
log10(average expression+0.01)
E
24h down-regulated genes in Oct4f/- ESCs + 4-OHT
21
7
597
4h down-regulated genes in Oct4-mAID ESCs + IAA
F
24h down-regulated genes in Oct4f/- ESCs + 4-OHT
15
13
8083
24h down-regulated genes in Oct4-mAID ESCs + IAA
